# Development of a New AMBER Force Field for Cysteine and Histidine Cadmium‐Binding Proteins and Its Validation Through QM/MM MD Simulations

**DOI:** 10.1002/jcc.70154

**Published:** 2025-06-13

**Authors:** Matteo Orlandi, Marina Macchiagodena, Piero Procacci, Fabrizio Carta, Claudiu T. Supuran, Marco Pagliai

**Affiliations:** ^1^ Dipartimento di Chimica “Ugo Schiff” Università degli Studi di Firenze Florence Italy; ^2^ Dipartimento di Neuroscienze, Psicologia, Area del Farmaco e Salute del Bambino, Sezione di Scienze Farmaceutiche Università degli Studi di Firenze Florence Italy

## Abstract

We developed and validated a novel force field in the context of the AMBER parameterization for the simulation of cadmium(II)‐binding proteins. The proposed force field takes into account the polarization effect produced by the central ion on its surroundings. The new polarized atomic charges for cysteine and histidine residues were derived based on the available structures of cadmium‐bearing proteins using QM calculations and QM/MM simulations. The developed force field was validated by performing molecular dynamics simulations on several cadmium(II)‐binding proteins. Our model preserves the tetra‐coordination of the metal site with remarkable stability, yielding mean distances between ${\mathrm{Cd}}^{2+}$ ion and S or N atoms of the binding residues in close agreement with experimental data.

## Graphical abstract

In the context of the AMBER parameterization, a new force field is developed and validated for the simulation of cadmium(II)‐binding proteins. The proposed force field takes into account the polarization effect produced by the central ion on its surroundings. The model preserves the tetra‐coordination of the metal site with remarkable stability.

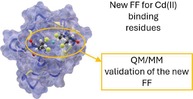

## 1 Introduction

Cadmium (Cd) is the chemical element with atomic number 48, belonging to the 12th group d‐block, located below zinc in the periodic table. Just like its neighbor, with whom it shares the full d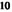 electronic configuration, cadmium shows a +2 oxidation state in almost all its compounds.

Cadmium is particularly known for its poisonous nature when interacting with living matter. Despite its utilization in rechargeable nickel‐cadmium batteries, it is recognized as carcinogenic and is known to cause damage to the kidneys, bones, cardiovascular, and reproductive systems. The Itai‐Itai disease is a particularly infamous illness that is caused by chronic cadmium exposure in humans.

From around 1910 up to the 1960s, the Jinzu River basin in Toyama [1] was contaminated by slag from a zinc mine, and subsequently, the Cd‐polluted water was employed to irrigate the rice paddies. This caused cadmium to accumulate in both the plants and the soil. Consequently, the inhabitants of the regions near the Jinzu River were exposed to Cd through the consumption of rice grown there.

The resulting long‐term cadmium exposure primarily affected the kidneys, with proximal tubular dysfunction found among the inhabitants of the river basin [2].

The Itai‐Itai disease can also cause bone injuries, consisting of a combination of osteomalacia and osteoporosis.

On the other hand, citing the words of Paracelsus: *“Omnia venenum sunt: Nec sine veneno quicquam existit. Dosis sola facit, ut venenum non fit” (Everything is poison, nothing exists without poison; only the dose makes it harmless)*, low concentrations of cadmium complexes are studied as innovative antibacterial and antitumoral agents nowadays [3, 4].

According to the HSAB (Hard Soft Acid Basis) theory [5], cadmium is considered a soft acid due to the high polarizability of its electronic cloud. Indeed, cadmium interacts mainly with soft bases, like sulfur. Spectroscopic and in vitro studies revealed that some cysteine‐rich zinc protein sites can easily exchange their native cofactor with cadmium, showing a greater affinity for the latter [6, 7]. In bioinorganic chemistry, cadmium finds application in the crystallization procedures. Cadmium salts are employed in the mother liquor to promote the crystallization process of a protein through the formation of coordination bonds with a negatively charged amino acid side chains. Many X‐ray structures, available in the Protein Data Bank (PDB) database [8], show cadmium ions coordinated by exterior surface side chains, where the metal executes a (nonbiological) structural function [9]. In the ion‐depleted environment of the ocean surface, colonial diatoms support their metabolic needs through a special kind of Carbonic Anhydrase: The ζ‐CA, which contains cadmium in the active site instead of zinc, as in human α‐CAs [10], has been speculated to maintain catalytic activity with both metals as an adaptation mechanism to the external environment [11].

Cadmium can substitute structural zinc in proteins [6], an essential cofactor that shapes and maintains the tridimensional arrangement and functionality of these macromolecules. In this regard, much evidence supports the idea that one of the possible causes of cadmium toxicity is related to the structural aberrations that this metal induces in the tertiary structure of host proteins, preventing proper folding [12] or disrupting protein activity [13, 14]. Regrettably, the lack of accurate classical force field approaches for the cadmium metal in proteins, exhibiting tetrahedral coordination, does not currently allow a reliable use of Molecular Dynamics (MD) simulations for the precise structural and dynamics characterization of the cadmium active sites, as well as the effect of cadmium substitution in zinc proteins.

This work, by focusing on tetrahedral cadmium protein complexes that are currently available in the PDB database through the MetalPDB interface [15], is aimed at engineering an accurate Force Field (FF) for classical MD simulations, that could reproduce the experimental metal‐ligand distances in a way that is accurate enough to enable the theoretical prediction of structural and functional modifications that can be induced by the presence of cadmium ion. To accomplish this task, we followed the same approach proposed by some of us in recent papers [16, 17], whereby the atomic charges of the coordinating residues are renormalized, using accurate ab initio calculations, to account for the polarization of a noncovalently bound metal.

In a recent work, different zinc‐tailored FFs, both bonded and non‐bonded types [18], were compared, and our approach was among the most successful in maintaining proper coordination patterns and reproducing experimental distances [19].

Therefore, as in References [16, 17], we relied on a non‐bonded strategy [20] to describe the interactions between the cadmium ion and the coordinating atoms. The resulting force field for cadmium proteins can be implemented with minor input modifications and no need for code upgrading in the most popular commercial and public molecular dynamics suites, and is essential to allow transient ligand or water exchanges in the coordination shell during the simulations. To counterbalance the small number of cadmium‐binding proteins identified as suitable for our purpose, we employed extensive QM/MM MD simulations as a reference and validation tool of the results obtained through classical trajectories.

## 2 Methodologies

### 2.1 Cadmium‐Proteins Dataset Assembly

To reparameterize the new coordinating amino acid residues, it was necessary to retrieve all the available cadmium metalloproteins from the metal‐PDB [15]. The metal‐PDB search parameters were set to (i) crystallographic resolution below 2.5 Å and (ii) “representative structure” only. We discarded all the structures that showed cadmium ions bound to the negatively charged side chains on the outer surface since, as mentioned in the “Introduction” section, these sites are artifacts of the crystallization procedure. The surviving structures, all exhibiting a tetracoordinated cadmium ion, constitute our training set for the force field parameterization of cadmium‐containing active sites in proteins.

The sites were then categorized depending on their coordination patterns, resulting in three groups: Four cysteine residues (4C), three cysteine and one histidine residue (3C1H), and a final group (AS) containing only two proteins with unique tetracoordination patterns, namely (i) Azurin in the *apo* form (PDB code 1AIZ) with two coordinating histidine residues (both of the N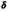 type), one cysteine and one glycine residue, and (ii) a Methionine Synthase (PDB code 1Q7Z) with three coordinating cysteines and one asparagine residue. This process allowed us to collect 10 non‐homologous protein structures, listed in Table 1. From these, 14 metal‐bearing active sites, including the metal ions and the coordinating residues, were identified (the structures with PDB code 1IPP, 1DCD, and 1VQ8 contain two, two and four active sites, respectively). The X‐ray structure 4C3D, exhibiting the lowest resolution in the training set, features a rather unusual and distorted quasi‐pyramidal tetracordination of the cadmium atom with disparate Cd‐S distances ranging from 2.29 to 2.55 Å. The structure 4C3D was hence further refined using the PDB‐redo web server [21].

**TABLE 1:** Cadmium‐binding proteins used for FF reparameterization (Re. is the resolution in Å and S.n. indicates the number of sites).

ID	Group	Re. (Å)	Function	Organism	S.n.	Chain
1IPP	3C1H	2.20	Transcription/DNA	Physarum	2	B
				polycephalum		
4C3D	3C1H	2.52	Viral Protein	Human respiratory	1	A
				syncytial virus A2		
1R0I	4C	1.50	Electron Transport	Clostridium	1	A
				pasteurianum		
1VQ8	4C	2.20	Ribosome	Haloarcula	4	1 3 U Z
				marismortui		
2JHF	4C	1.00	Oxidoreductase	Equus	1	B
				caballus		
2PZI	4C	2.40	Transferase	Mycobacterium	1	B
				tuberculosis		
1FE0	4C	1.75	Metal Transport	Homo	1	A
				sapiens		
1DCD	4C	2.00	Electron Transport	Megalodesulfovibrio	1	B
				gigas		
1AIZ	AS	1.80	Electron Transport	Achromobacter	1	A
				denitrificans		
1Q7Z	AS	1.70	Transferase	Thermotoga	1	B
				maritima		

### 2.2 Ab Initio Determination of Atomic Charges in Cadmium(II)‐Binding Cysteinate and Histidine Residues

**FIGURE 1:**
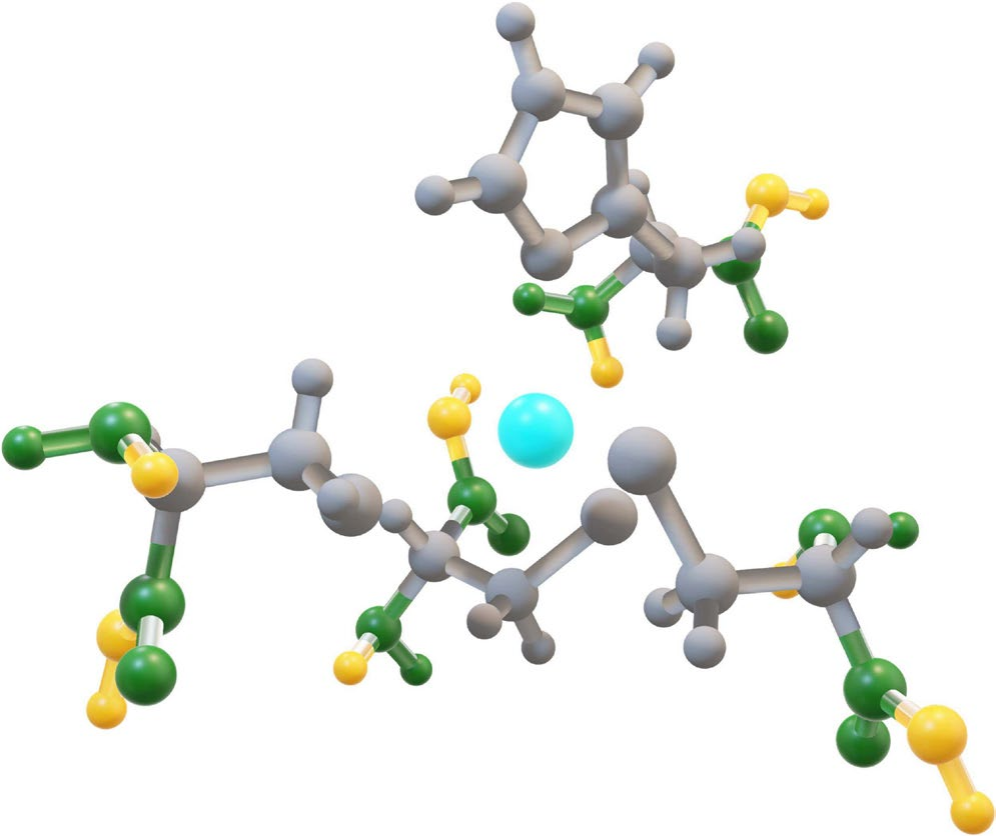
Example of the cluster used for the fitting of the side‐chains atomic charges. The green‐colored atoms represent the terminal carbonyl and amine functional groups, whose charges were constrained to the standard AMBER values. In yellow, the hydroxyl and hydrogen saturating atoms are shown, whose atomic charges were left unrestrained. In cyan and gray, the cadmium ion and the atoms of the reparameterized residues are shown, respectively.

For each of the 14 catalytic sites extracted from the PDB structure of Table 1, we first isolated the coordinates of the metal and of the binding residues. The amino acid ligands were saturated with a hydrogen atom (‐H) at the N‐terminus extremity, and with a hydroxyl group (‐OH) at the carbonyl one. Once the 14 extracted and saturated active sites had been prepared, ab initio quantum chemical calculations were performed relying on the Merz–Kollman restrained electrostatic potential (RESP) scheme [22] to obtain atomic charges. In the RESP fitting procedure, the atomic charge of the cadmium ion was set to +2e, while the terminal carbonyl (C=O) and amino groups (N‐H) were constrained to the AMBER FF [23, 24] standard values to ensure force field compatibility. In addition, the atomic charges of the hydroxyl and the hydrogen saturating atoms were left unrestrained (Figure 1). The charges of the unique glycine and asparagine cadmium‐binding residues referring to the AS group, including Azurin (PDB code A1IZ) and Methionine Synthase (PDB code 1QTZ), were constrained to the standard AMBER values.

The total charge of the reparameterized residues was fixed to the integer value of −1e for cysteinate and 0e for histidine.

For each of the 14 cadmium (II) sites in the training set, RESP atomic charges were computed using the CP2K suite of programs [25], with the Quickstep module [26]. To this end, Density Functional Theory (DFT) single‐point calculations were carried out using the PBE exchange and correlation functional [27, 28] in conjunction with the hybrid Gaussian and plane wave method [29] with the DZVP‐MOLOPT optimized basis set [30], modeling the core electrons of each atom through compatible Goedecker‐Teter‐Hutter pseudopotentials [31, 32, 33], and describing electron density with a plane‐wave expansion of 400 Ry.

The system was treated as isolated, employing the Martyna et al. [34] algorithm with a cubic simulation box of 25 Å side length. The single‐point CP2K calculations were performed using the PDB coordinates of the active sites as described in the section “Cadmium‐proteins Dataset Assembly”.

In the RESP calculation, all grid points within a given spherical shell (defined by a minimal radius $r_{\min}$ equal to van der Waals radius and a maximal radius $r_{\max}$ equal to the van der Waals radius multiplied by 3) surrounding each atom were included in the fitting.

The calculated charges were averaged into 3 groups, depending on their coordination pattern. The final atomic charges for each residue atom were then determined through a weighted average based on the relative occurrence of each coordination pattern in the database.

### 2.3 Potential Energy Surface Scan of Cadmium‐Water Complex

Ab initio soft Potential Energy Surface (PES) scan calculations were carried out through Gaussian09 [35] software. The def‐TZVP [36] basis set was selected to describe the wave function of the system, which is composed of a cadmium ion and a water molecule. The scan was performed along the Cd‐O interatomic distance in the range 1.8–6.0 Å with increments of 0.1 Å, followed by a geometric optimization of the water internal coordinates at each step. The reference calculation was performed at the Møller–Plesset perturbation theory (MP2) [37] level, taking into account the Basis Set Superposition Error (BSSE).

The classical PES scan was obtained by extracting the MP2‐optimized system configurations from the Gaussian09 output, which were then employed for single‐point Molecular Mechanics (MM) potential energy calculations through the GROMACS software [38]. Cadmium was described using the compromise (CM) non‐bonded parameters of Li et al. [39] and the SPC/E [40] model was employed for water. A cut‐off of 1 nm was imposed on the van der Waals and electrostatic contributions to the potential energy.

### 2.4 Classical Molecular Dynamics Simulations

Classical Molecular Dynamics (MD) simulations of cadmium‐binding proteins were carried out in a cubic box with periodic boundary conditions, whose side length was chosen so that the minimum distance between protein atoms belonging to neighboring replicas was larger than 20 Å in any direction. Proteins were explicitly solvated using the SPC/E water model [40, 41] at the standard density. The starting configuration was generated using the standard GROMACS ancillary tools [38]. In all MD calculations, we used the AMBER99SB‐ILDN force field [42] patched with our newly developed residues and the 12‐6 Lennard‐Jones (LJ) CM parameters for the cadmium divalent ion from Li et al. [39].

The systems were initially minimized at 0 K with a steepest descent algorithm and subsequently heated to 298.15 K in the NPT (isothermal isobaric) ensemble setting P=1 atm by means of a Parrinello‐Rahman barostat [43] and velocity rescaling algorithm [44] with an integration time step of 0.1 fs and a pressure coupling constant $\tau_{P}$ of 0.1 ps for 1000 ps.

The production run was carried out in the NPT ensemble for 50 ns, imposing rigid constraints only on the X‐H bonds (with X being any heavy atom) through the LINCS [45] algorithm using a time step of 2.0 fs. Electrostatic interactions were treated using the PME [46] method with a grid spacing of 1.2 Å and a spline interpolation of order 4. The nonbonded 1–4 interactions involved in a proper torsion were scaled by the standard AMBER fudge factors (0.8333 and 0.5 for Coulomb and Lennard‐Jones, respectively). The simulations and the trajectory analysis were performed using the GROMACS 2022.3 suite of programs [38].

The crystal structure of each protein was considered as the reference for the Root Mean Square Deviation (RMSD) calculations.

### 2.5 Quantum Mechanics/Molecular Mechanics Molecular Dynamics Simulations

QM/MM MD simulations are employed to study the time evolution of a small region of interest belonging to an extended molecular system through the principles of quantum mechanics, while molecular mechanics is concurrently used to solve the equations of motion of what remains outside the QM subsystem. We chose the CP2K/GROMACS [25, 38] interface to carry out the canonical QM/MM MD simulations. Three QM/MM MD simulations were performed on selected proteins with three different coordination patterns: 1R0I (4C), 1IPP (C3H1), and 2L1O (C2H2) [47].

**FIGURE 2:**
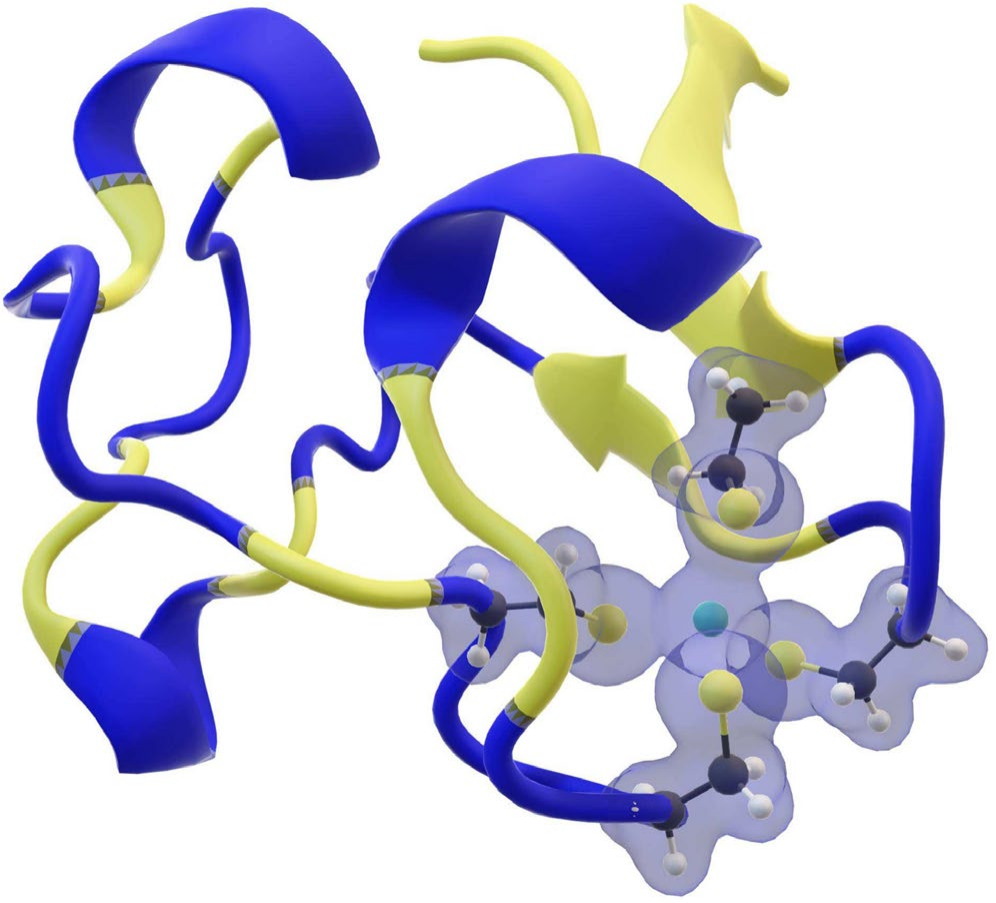
Representation of the QM subsystem electron density belonging to the 1R0I protein (obtained at the same QM/MM level of theory of the MDs) consisting in 4 cysteine residues (up to each Cα) and the cadmium ion.

The latter, 2L1O protein, was excluded from the dataset (Table 1), since it was obtained through NMR and the cadmium ion was inserted after structure minimization of the apoprotein, leading to unrealistic metal‐ligand interatomic distances. For the MM part of the systems, we relied on the AMBER99SB‐ILDN FF [42]. The QM region features the metal ion and all the atoms belonging to the coordinating amino acid side chains, including the Cα and Hα atoms (see Figure 2). All the QM atoms were described performing DFT calculations with the PBE exchange and correlation functional, combined with the DZVP‐MOLOPT basis set [30]. The core electrons of each atom were modeled through the Goedecker‐Teter‐Hutter (GTH) pseudopotentials [31, 32, 33] (the SR version was used for Cd), while the electron density was described with a plane‐wave expansion of 450 Ry. Grimme D3 dispersion corrections [48] were also included, with a cut‐off set to 16 Å.

The QM box dimensions were automatically set by the interface.

No classical charge redistribution was attempted but we verified that the spreading of the excess of negative charge, that arises from the substitution of the MM region with the QM one, on the neighboring MM atoms (the amidic nitrogen and the carbonyl carbon for each residue) did not affect Cd ligand interatomic distances [49]. Moreover, the CP2K/GROMACS interface relies on the so‐called Gaussian Expansion of the Electrostatic Potential (GEEP) approach [50] that smears the classic atomic charges at the QM/MM interface, preventing the electronic spill‐out problem from the QM region. The GEEP electrostatic embedding [50] was enforced using 12 Gaussian functions for the expansion of the MM potential. For each amino acid, 2 saturating hydrogen atoms were added, as link atoms, to the QM subsystems by the CP2K/GROMACS interface to complete their valence, as shown in Figure 2.

The starting configurations for the 3 QM/MM MD simulations were extracted from the final step of the corresponding classical NPT production MD simulations, maintaining the same periodic boundary conditions (PBC) box dimensions. The QM/MM systems were initially equilibrated in the NVT ensemble for 2 ps, with a time step of 0.5 fs. All the other classical simulation parameters were left unchanged from the previous MD production runs, except for the barostat. The equilibrated configurations obtained were selected as the starting points for the 10 ps NVT QM/MM production runs, employing the same time step of 0.5 fs. All the QM/MM MD simulations were launched on the DCGP partition of Cineca's LEONARDO supercomputer cluster, using a hybrid 16 MPI/7 openMP scheme. Each calculation ran for at least 20 h on 2 nodes, equipped with a total of 224 CPU cores.

## 3 Results and Discussion

### 3.1 Potential Energy Surface Scan

As suggested in Reference [19], where FFs for zinc‐binding proteins were thoroughly benchmarked, the combination of the FF parameters of the coordinating residues from Macchiagodena et al. [16] with the CM LJ 12‐6 ${\mathrm{Zn}}^{2+}$ parameters proposed by Li et al. [39] (Li12‐6) led to enhanced stability in the simulations of the tetrahedral zinc ions, even when one of the residues was not among the four for which side‐chain atomic charges were reparameterized. Since the Li12‐6 parameters are also available for the cadmium divalent ion [39], such an approach could fruitful in the construction of a modified AMBER FF for cadmium‐containing proteins.

As done in Reference [19] for the ${\mathrm{Zn}}^{2+}$ ion, here we preliminarily compare the energy of the ${\mathrm{Cd}}^{2+}$‐water obtained with the ${\mathrm{Cd}}^{2+}$ ion, using Li12‐6 parameters, and the SPC/E model to that derived using ab initio MP2 calculations. This comparison is important as water tends to interfere with the metal coordination environment in classical MD simulations, sometimes disrupting the tetrahedral arrangements [19], if the energetic balance between the ${\mathrm{Cd}}^{2+}$‐water and ${\mathrm{Cd}}^{2+}$‐residues interaction is not optimized.

**FIGURE 3:**
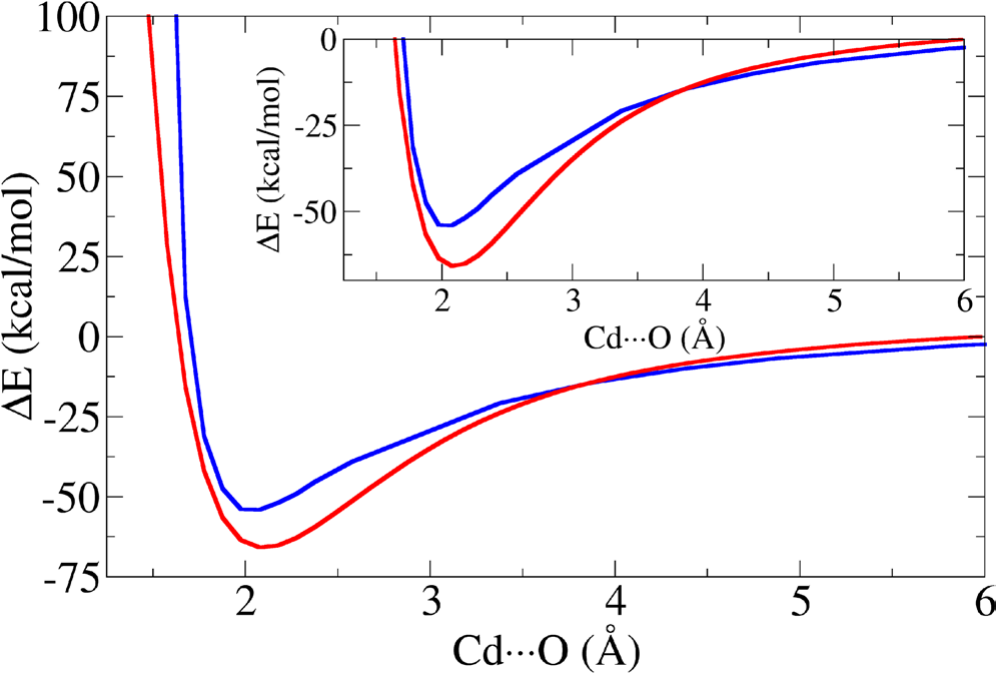
Potential energy surface scan and relative focus around the minimum for the ${\mathrm{Cd}}^{2+}$‐water interaction obtained using the ${\mathrm{Cd}}^{2+}$ Li12‐6 LJ parameters with SPC/E water model (blue curve) and MP2 calculations (red curve).

**FIGURE 4:**
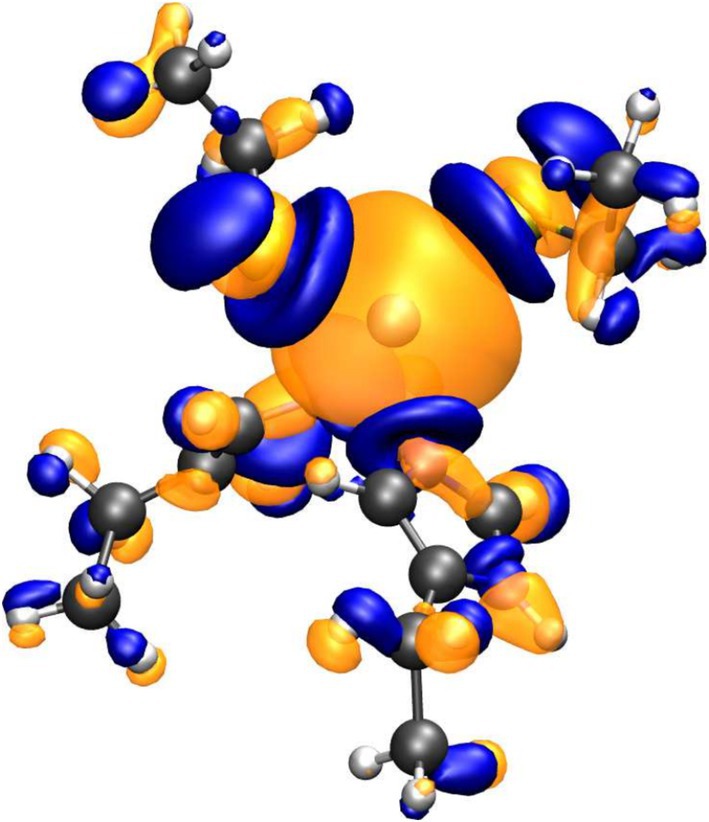
Representation of the electron density resulting from the subtraction of the density of the full QM/MM system (quantum region includes cadmium and binding side chains) minus that of the QM/MM system where the cadmium is assumed to be classical. In blue, the negative regions are depicted, while the positive ones are shown in orange.

Results of such a comparison are shown in Figure 3. As it can be seen, the combined 12‐6 LJ and Coulomb potential energy functions, using the Li12‐6 LJ parameters for the cadmium ion, approximate the reference MP2 PES, showing comparable accuracy to the corresponding 12‐6 LJ parameterization for ${\mathrm{Zn}}^{2+}$ with TIP3P (see Figure 4 of Reference [19]). In the range spanning 1.8–3.0 Å. The classical MM Li12‐6 model for ${\mathrm{Cd}}^{2+}$ reproduces the correct MP2 equilibrium distance, placing it exactly at 2.076 Å, but it underestimates its depth of about 11 kcal/mol. Significant differences are also seen for Cd‐O distances below 1.7 Å, where the classical potential rises more sharply compared to the MP2 counterpart, and in the range 3.0–6.0 Å, where the derivative of the 12‐6 LJ and Coulomb parameterization decreases more rapidly than that obtained with MP2. However, these discrepancies, likely attributable to the limitation of the simple 12‐6 Lennard‐Jones function for the dispersive‐repulsive interactions should be of minor importance when simulating cadmium proteins in standard conditions (i.e., with small oscillation around the minimum).

The results obtained using the Li12‐6 model for the ${\mathrm{Cd}}^{2+}$‐water interaction fully confirms the finding of Reference [19], where the same classical PES for the zinc‐water interaction using the Li12‐6 model was compared to the MP2 counterpart. Thus this simple classical ${\mathrm{Cd}}^{2+}$ parameterization was chosen for the development of our FF for cadmium‐bound polarized residues in SPC/E solvated proteins.

### 3.2 The Force Field for Cadmium(II)‐Binding Cysteinate (CYC) and Histidine (HEC and HDC) Residues

In Figure 4, we show the differential electron density Δρ obtained in single point CP2K/GROMACS QM/MM calculations on the structure 1R0I by subtracting (i) the density ($\rho [{\text{Cd}}^{2+}]$) obtained by including the side chains only in the QM region while leaving the cadmium ion in the MM subset, to (ii) the density ($\rho [{\text{Cd}}_{\text{QM}}]$) calculated including the cadmium atom and binding side‐chains in the QM region, that is, $\Delta \rho =\rho [{\text{Cd}}_{\text{QM}}]-\rho [{\text{Cd}}^{2+}]$. Such electron density difference provides a detailed picture of the polarization effects of the cadmium divalent point charge on the coordinating residues. Inspection of Figure 4 shows that the classical treatment of the cadmium cation strongly affects the density on the side chains, showing significant polarization effects. In particular, the blue regions on the binding sulfur and nitrogen atoms mark a significant increase in electron density when cadmium is treated as a simple point charge of +2e. Such a result justifies the use of strongly polarized charges on the cadmium‐binding groups in a purely classical treatment. Moreover, as shown in the differential density of Figure 4, the spatial extent of the electron polarization and diffusion on the cadmium‐binding atoms suggests that the effective van der Waals radius of the atoms non‐covalently coordinating the central ion should be chosen larger compared to the standard radius, referring to the interactions with less polarizing counterparts.

**FIGURE 5:**
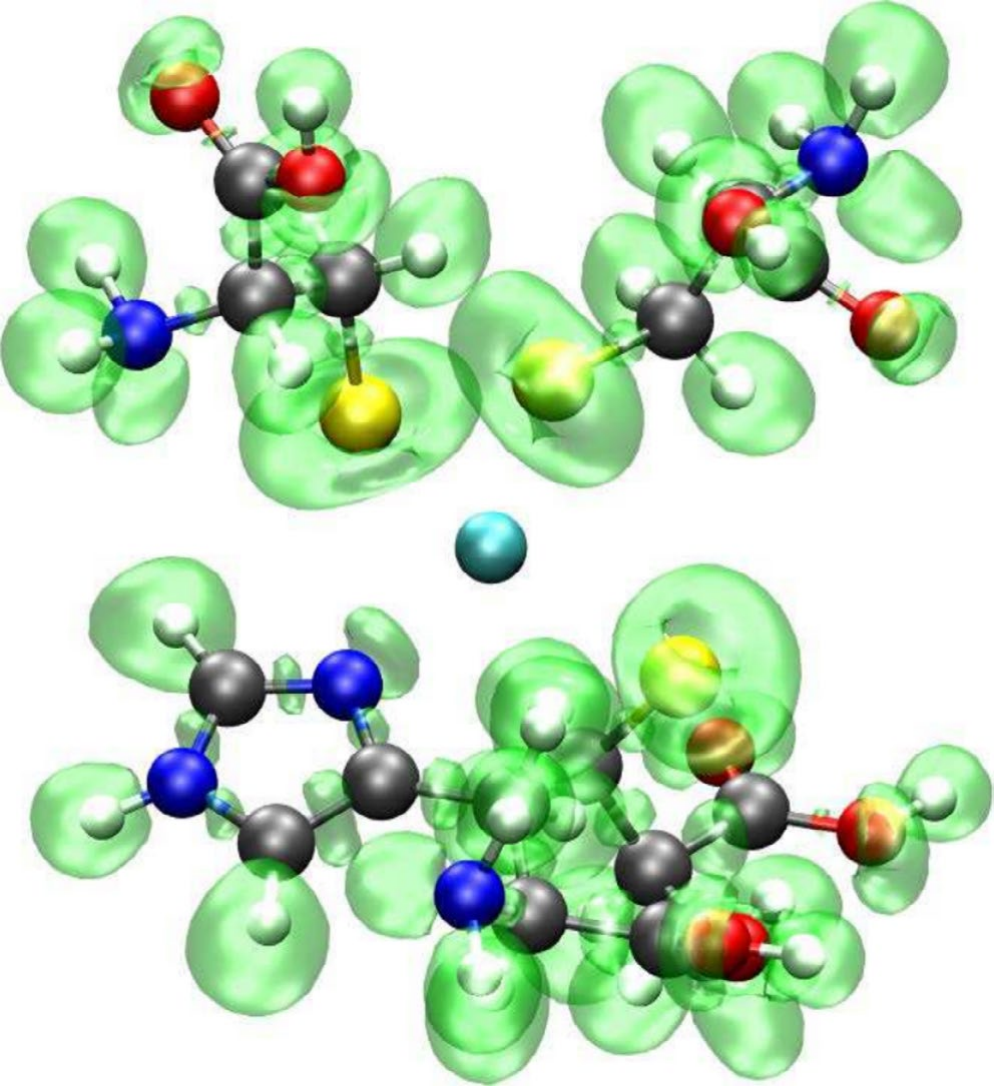
Representation of the electron localization functions on the active site of the 1IPP structure (C3H1 coordination) obtained using a single‐point CP2K calculation.

The use of a well‐tuned *non‐bonded* polarized classical model for the description of the cadmium in proteins is further justified in the Figure 5, where we show the Electron Localized Function (ELF) [51] mapping the electron pair probability on the active site of the 1IPP (C3H1 coordination) structure obtained using a single‐point CP2K calculation. The lack of ELFs between the cadmium ion and binding S or N atoms is a clear indication of the non‐covalent nature of the interaction between cadmium and the coordinating atoms.

The indications from the differential electron density Δρ and the ELF picture shown in Figures 4 and 5 translate into the adoption of a RESP fitting protocol (see “Methods” section for details) for the atomic charges on the residues, whereby we fix the +2e charge on the cadmium cation while adjusting the side‐chain charges only of the coordinating residues to reproduce the QM electrostatic potential. The calculated RESP charges on each of the 14 metal‐bearing sites in Table 1 were averaged into three groups, depending on their coordination pattern. The final atomic charges for each residue atom were then determined through a weighted average, reflecting the relative occurrence of each coordination pattern across the dataset. The results of the RESP calculation are collected in Tables S1–S3 of the Supporting Information (SI) for the cysteine and histidine, of type δ and ϵ, residues. It can be noted that in all cases, the atomic charges on the cadmium coordinating atoms, whether on nitrogen or sulfur atoms, are strongly more negative compared to the standard non‐polarized AMBER charges. Tables S1–S3 also show that when the cadmium‐induced RESP charges of the present study are matched by atom label to the polarized RESP charges induced by the zinc atom, as computed by Macchiagodena et al. [16], using the same QM approach, they are found consistently more polarized, ranging from an excess negative charge of 3% on the sulfur atom, to 15% and 30% excess on the nitrogen atoms compared to the polarized zinc‐binding counterpart, hence reflecting a stronger polarization effect of the softer and diffuse cadmium divalent ion. The reparametrized cadmium‐binding residues are indicated with CYC for the cysteine and with HDC, HEC, for ϵ and δ histidine residues, respectively.

Once the sets of atomic polarized charges were determined for the new cadmium‐binding amino acidic residues, we managed to assign new σ parameters to the Cd coordinating atoms while leaving the corresponding ϵ parameters unchanged (i.e., identical to the AMBER standard values) as done in Reference [16]. As extensively discussed in the previous section, the cadmium σ and ϵ parameters are assumed to be those reported by Li et al. [39] (CM) with no modification whatsoever. Thus, the tuning of the LJ potential in modeling the cadmium‐protein interaction involves only the σ parameters of the cadmium‐binding atoms (sulfur or nitrogen), recovering the overall Lennard‐Jones parameterization from the AMBER standard mixing rules for σ and ϵ. After multiple trials and adjustments based on both experimental and QM/MM structural data as reference, through several classical MD runs on the protein collection of Table 1, we assigned a σ value of 4.400 Å to the sulfur, which was renamed as SC. The 23.5% increase from its standard AMBER FF value (see Table S4) is justified by a substantial 16.9% increase of atomic charge on the same atom. For histidine cadmium‐coordinating nitrogen, we introduced two new atom types, namely (i) NC4, representing Nδ with a σ=4.200 Å, increased by 29.2% compared to the AMBER standard in histidine residues, and (ii) NC5 for the Nϵ variant with a σ=3.950 Å, increased by 21.5% compared to AMBER standard. Also, in this case, the large van der Waals radius for the σ parameter of nitrogen reflects an increase in atomic size due to the excess electrons found via RESP on the polarized cadmium‐binding nitrogen, compared to the non‐polarized (AMBER standard) counterpart where the charges on the nitrogen are less than half.

### 3.3 Classical and QM/MM MD Validation of the New FF

In this Section, we report and discuss the results of the classical MD simulations on several proteins, mostly taken from the Table 1, obtained with the new force field and with the standard AMBER force field. For each simulated protein, we extracted at a regular interval during the 50 ns trajectories, the time‐dependent interatomic distances between the cadmium ion and the coordinating atoms, as well as the RMSD fluctuations of the protein backbone, the coordinating atoms, and the coordinating residues, both along with the metal, using the corresponding experimental structure as a reference.

In total, we performed 20 classical MD simulations on cadmium‐bearing proteins, for a cumulative simulation time of 1 μs. In Table 2, we report the mean Cd‐S and Cd‐N distances recorded during these simulations with our new and the AMBER standard FF, compared to the experimental counterpart and to QM/MM CP2K calculations (when available). For each protein entry in the table (identified via the PDB code), Cd‐S and Cd‐N distances were averaged over all similar coordinating bonds in the protein. For the cases of the MD simulation of 1DCD and 1IPP, the Cd‐S and Cd‐N average distances were taken over the two cadmium sites. For the complex 1VQ8, we performed two independent simulations for chain 1 (ribosomal protein L39E) and chain 3 (ribosomal protein L11P) both belonging to the group 4C (see Table 1). The results show a remarkable overall agreement of the Cd‐S and Cd‐N average distances with the X‐ray counterpart when the new FF with polarized residues is adopted. In addition, the small standard deviations of the Cd‐X distances are an indication of the stability of the tetracoordination. Conversely, when the AMBER standard FF is adopted, Cd‐X distances are significantly shorter than the experimental counterpart, and, notably, in the two proteins belonging to the 3C1H group, (4C3D and 1IPP), tetra‐coordination is lost due to histidine detachment. The NMR structure 2L1O, referring to Thaliana Superman zinc finger (2C2H group) [47], was not part of the set for RESP charges derivation, due to the uncertainty on the Cd‐X experimental distances, with standard deviations (computed from the 20 NMR models reported in 2L1O pdb) that are two‐fold and four‐fold for Cd‐S and Cd‐N, respectively, when compared to the mean standard deviation in the dataset. At variance with what was observed when using the AMBER standard FF, the simulation of the 2L1O zinc finger using the new parameterization for cadmium‐binding CYS and HIS, exhibits a stable tetracoordination with average Cd‐S and Cd‐N distances and corresponding standard deviations that are similar to those observed in the other proteins belonging to the original set. Moreover, the calculated average interatomic distances are in close agreement with those obtained through QM/MM MD simulation. These evidences provide a convincing validation of the proposed parameterization. Considering the complexity of the conformational landscape of 2L1O and its force field‐dependent fragility, an additional 3 replicas of 50 ns were conducted to further validate our new FF's reliability (RMSD fluctuations reported in the SI). We verified that the transition to the TIP3P water model does not affect the accuracy of our new FF, and its performances were proven over a longer simulation time scale (400 ns) for two selected proteins: 1R0I and 1IPP (Figures S1 to S7 of SI).

**TABLE 2:** Comparison of the mean interatomic Cd‐S and Cd‐N distance (in Å) obtained through classical MD using our new FF, the AMBER standard FF, QM/MM MD (when performed) and X‐ray structure reference.

Protein	d(Cd‐X)	New FF	AMBER FF	QM/MM MD	EXP
1DCD	Cd‐S(8)	2.53 ± 0.05	2.19 ± 0.04		2.54
1FE0	Cd‐S(4)	2.53 ± 0.06	2.19 ± 0.04		2.48
1IPP	Cd‐S(6)	2.52 ± 0.05	2.23 ± 0.07	2.52 ± 0.06	2.64
	Cd‐N(2)	2.37 ± 0.04	4.77 ± 3.00	2.40 ± 0.09	2.39
1R0I	Cd‐S(4)	2.53 ± 0.05	2.20 ± 0.04	2.59 ± 0.08	2.52
1VQ8_1	Cd‐S(4)	2.52 ± 0.05	2.19 ± 0.04		2.53
1VQ8_3	Cd‐S(4)	2.53 ± 0.05	2.27 ± 0.06		2.60
2JHF	Cd‐S(4)	2.51 ± 0.05	2.21 ± 0.06		2.55
2PZI	Cd‐S(4)	2.52 ± 0.05	2.19 ± 0.04		2.59
4C3D	Cd‐S(3)	2.53 ± 0.06	2.27 ± 0.06		2.52
	Cd‐N(1)	2.27 ± 0.04	5.99 ± 2.55		2.26
2L1O	Cd‐S(2)	2.55 ± 0.08	2.24 ± 0.05	2.49 ± 0.06	2.67 ± 0.11
	Cd‐N(2)	2.27 ± 0.04	4.26 ± 2.20	2.39 ± 0.09	2.31 ± 0.24

**FIGURE 6:**
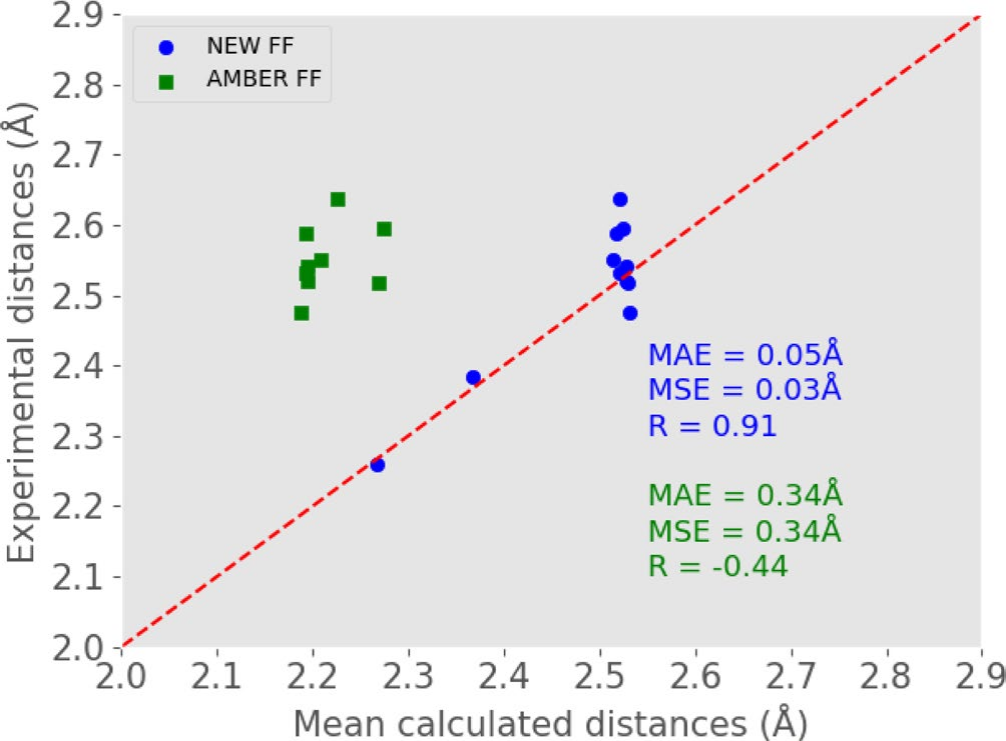
Comparison between experimental Cd‐X interatomic distances and those obtained by MD simulations using our NEW FF (blue) and standard AMBER FF (green). Three AMBER standard values were out of range and are not shown in this graph. MAE: Mean absolute error; MSE: Mean signed error; R: Pearson correlation coefficient.

The performance of the new FF for cadmium‐bearing proteins can be best appreciated from Figure 6, where we report the correlation diagram between experimental Cd‐X distances and MD‐derived counterpart. The Pearson correlation coefficient, computed over the entire set of Cd‐X distances, hits 0.91 when the new FF is used, with a mean absolute error of just 0.05 Å. When using the standard AMBER FF, experimental and MD‐derived distances are found to be *anti‐correlated*, even excluding from the set the three Cd‐N distances referring to histidine residues that lost coordination during the simulation (namely for 2L1O, 1IPP, 4C3D). Moreover, the AMBER standard systematically and significantly underestimates all Cd‐X distances, yielding identical mean absolute and signed error.

**FIGURE 7:**
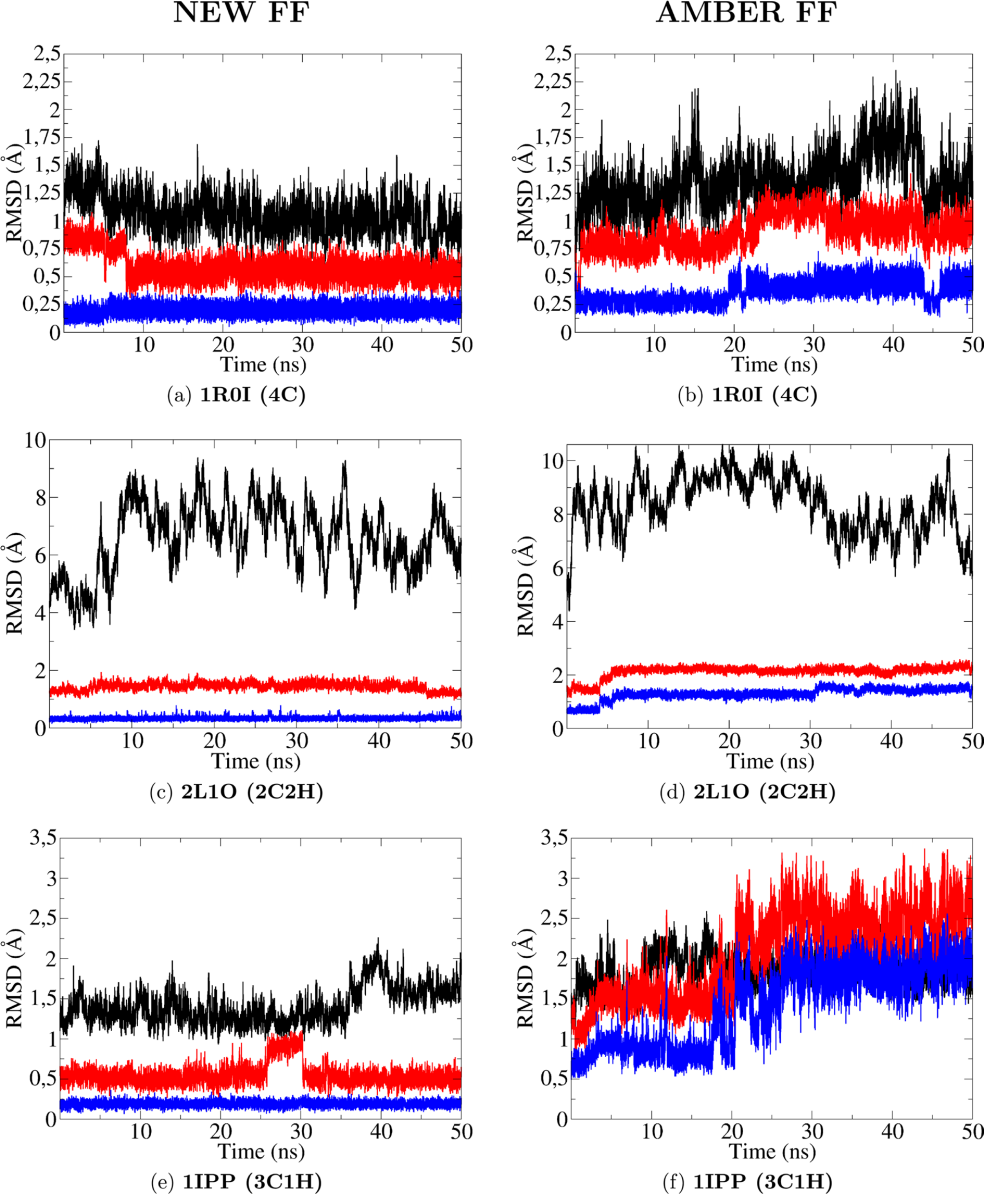
From top to bottom: **1R0I** (4C, panels a–b), **2L1O** (2C2H, 2 Nϵ, panels c–d) and **1IPP** (3C1H, Nδ, panels e–f) RMSD fluctuations: In blue: Cd‐coordinating atoms, in red Cd‐coordinating residues and black backbone. On the left column, the results obtained through our new FF are shown, whereas on the right column, those of the AMBER FF are shown.

As representative examples, in Figure 7 we report the RMSD results for the simulations for the cadmium proteins 1R01, 2L1O, and 1IPP with the new FF (left panels, Figure 7a,c,e) and the standard AMBER force field (right panels, Figure 7b,d,f). RMSDs for the remaining seven proteins are reported in the SI (Figures S8 to S11). The time record of the RMSDs (coordinating atoms, coordinating residues, and backbone) provides a valuable test for assessing the stability of the coordination shell, as well as that of the folded state.

**FIGURE 8:**
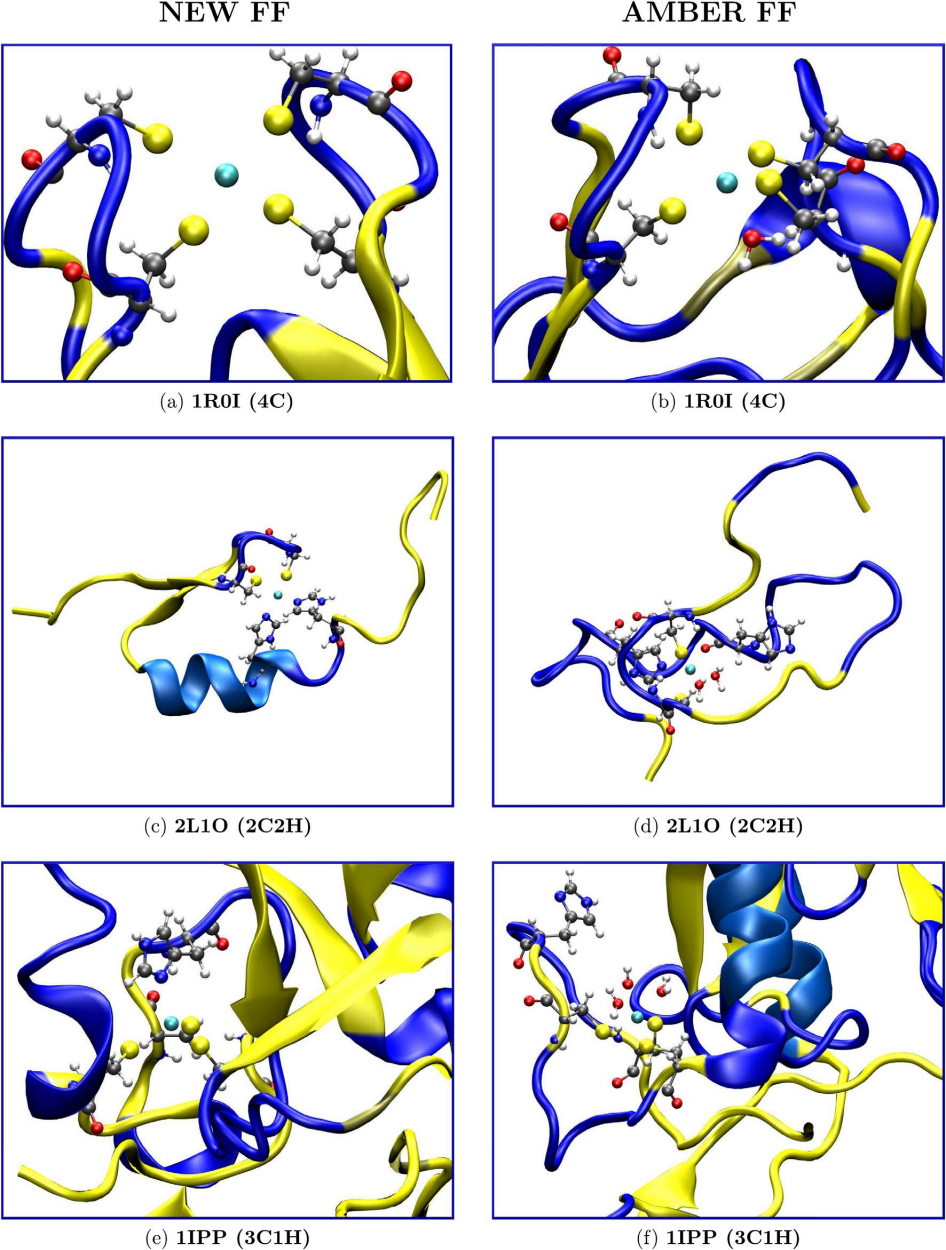
From top to bottom: **1R0I** (4C, panels a–b), **2L1O** (2C2H, 2 Nϵ, panels c–d) and **1IPP** (3C1H, Nδ, panels e–f) final snapshots extracted from the MD simulations. On the left column, the results obtained through our new FF are shown, whereas on the right column, those of the AMBER FF are shown.

In all three reported simulations, we observe that the new FF leads to structures preserving the original arrangements of the cadmium site (see Figure 8a,c,e), of the surrounding residues, and of the protein fold. In 1R01 and 1IPP, the RMSD deviation for the four Cd‐binding sulfur atoms is minimal, as are those of the coordinating residues and the full backbone. Nevertheless, transient events, such as the ephemeral insertion of water molecules, are still possible thanks to the non‐bonded approach. In this respect, the sudden jump around the beginning of the simulation of the coordinating side‐chains RMSD (red line) in the simulation of 4C3D (left panels, Figure S9a), reflects a transient change in the coordination sphere, caused by the interference of a water molecule. The non‐bonded approach can thus guarantee a certain degree of flexibility in the simulations, potentially enabling our FF to handle more challenging environments, like metalloenzymes, whose coordination sphere might be incomplete and accessible to exogenous molecules. In this latter case, in particular, the allowance for a dynamic ligand exchange can play a crucial role in the realistic characterization of these systems. In the 2L1O simulation reported in the central panel of Figures 7c and 8c, the reference structure corresponds to model 1 in the PBD file. In this case, while tetracoordination is very well preserved as assessed by the RMSD of the binding atoms (on the order of 0.2–0.3 Å) and of the binding side‐chain atoms (of the order of ≃1 Å), the RMSD of the backbone atoms is rather large, reflecting the random coil behavior of the N‐terminal domain 28‐38 of the zinc finger.

In Figure 7b,d,f, we report the RMSDs obtained with the standard AMBER FF. In all three cases, the AMBER standard approach is unable to preserve the tetrahedral arrangement of the cadmium divalent ion. Tetracoordination is disrupted after a few ns in the case of the 2L1O and 1IPP protein. In 1R0I, a water molecule enters the coordination sphere after ≃20 ns, producing a stable trigonal bipyramid structure lasting up to the end of the simulation. In the case of the zinc finger 2L1O, the loss of tetracoordination right at the start of the simulation induces a catastrophic collapse of the fold of the C‐terminus 1–28, as shown in the zinc finger final structure reported in the central panel of Figure 8d. In both 1IPP and 2L1O simulations, the disruption of the tetracoordination is triggered by the detachment of the coordinating histidine side‐chain. We recall that in the AMBER standard, the charge on the coordinating nitrogen of δ (HID) and ϵ type (HIE) is −0.57 e and −0.54 e, respectively compared to values of ≃−1.30 e in the new force field (see Table S3) with polarized side chains. The low charge on the N atoms in the AMBER standard entails a severe underestimation of the N‐Cd Coulomb interactions, favoring the substitution of the coordinating histidine nitrogen with the oxygen of a water molecule.

**FIGURE 9:**
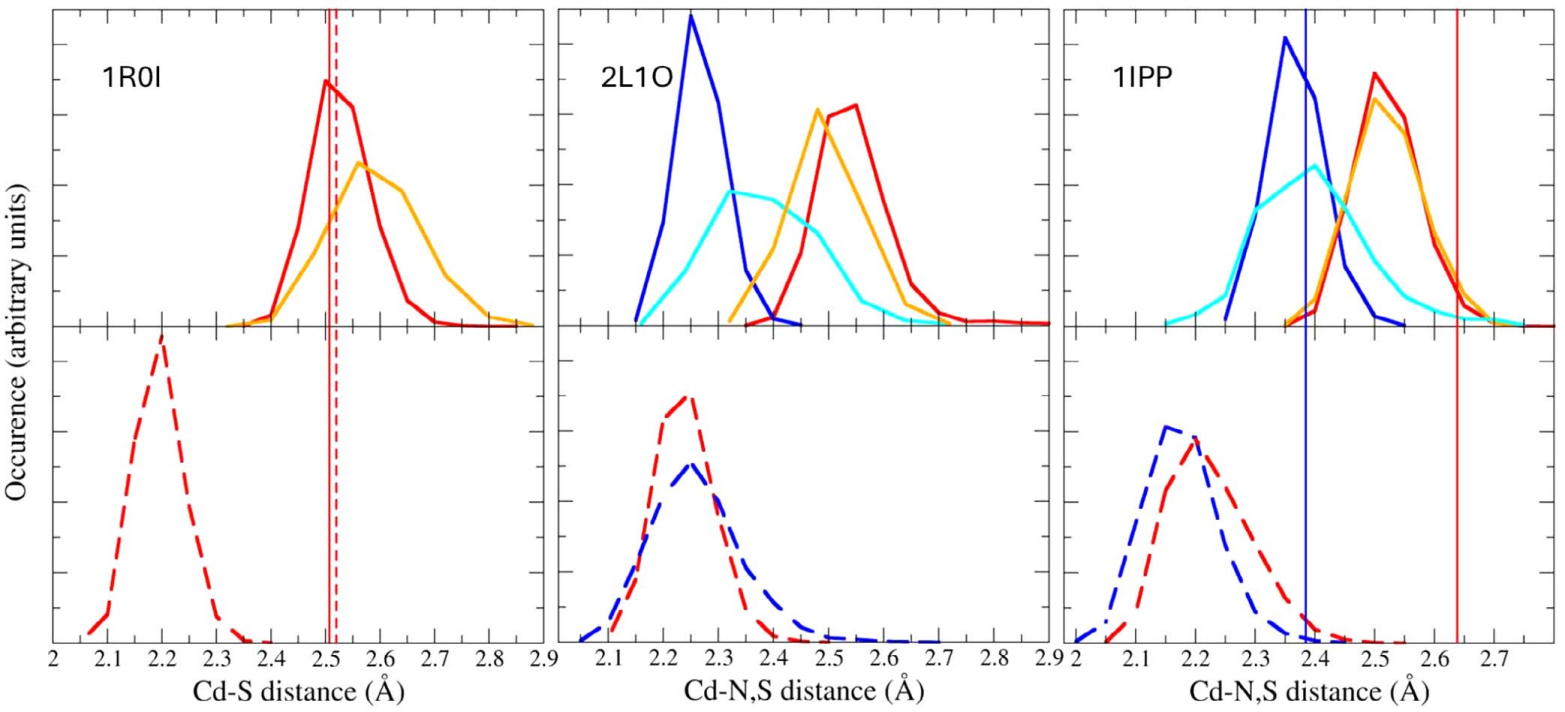
Interatomic distance distributions obtained through MDs of the proteins 1R0I, 2L1O, and 1IPP. In the top and bottom panels, we report the results obtained with the new (solid line) and AMBER standard FFs (dashed line), respectively. The red and blue colors refer to the Cd‐S and Cd‐N distance distribution, respectively. The solid vertical lines are the X‐ray experimental distances, while the dashed ones indicate the PDB‐redo analogous distances. In red, we report the corresponding Cd‐S distances. In cyan and orange, the Cd‐N and Cd‐S distance distributions resulting from the 10 ps QM/MM simulations are presented.

In Figure 9, we finally show the probability distribution of the Cd‐X distances in the three representative proteins 1R0I, 1IPP, 2L1O obtained from classical MD simulations (50 ns) and the corresponding QM/MM simulations (for remaining proteins the results are reported in Figures S12 to S14 of SI). For 2L1O and 1IPP proteins, the interatomic Cd‐N distance distributions of the detached histidine residues are not reported. Vertical lines on the plot mark the experimental distances. In general, we note that QM/MM Cd‐S distribution closely follows that obtained with the classical MD relying on the new FF, in the position of the peak (which is close to the experimental X‐ray value) as well as in the spread and quasi‐Gaussian shape. The QM/MM distribution is slightly wider and up‐shifted compared to the classical one (new FF) for the Cd‐N distances, reflecting a slightly weaker ionic bond as probed in the QM region of the QM/MM simulation for the nitrogen‐cadmium interactions. When using the AMBER standard force field in classical MD simulations, both the Cd‐S and Cd‐N distances are found to be strongly underestimated, with distributions peaking at least 0.3 Å away from the QM/MM peak and the corresponding experimental values.

## 4 Conclusions

Over the years, evidence has been gathered about cadmium toxicity when interacting with the living matter, but the mechanism through which cadmium exerts its poisonous nature against proteins remains still unclear.

Lacking of a reliable parametrization for cadmium‐amino acids interactions has so far hampered the widespread use of classical molecular dynamics simulation to study structural and dynamical properties of cadmium‐bearing proteins at the atomistic level. In an attempt to bridge this divide, in this work, we developed a classical FF for cadmium and cadmium‐binding residues that explicitly takes into account the persistent polarization effect produced by the central ion on its surroundings. To this end, we adopted a procedure for the reparameterization of Cd ion in proteins that has already been used in the redefinition of the AMBER FF for zinc‐binding proteins [52] and that is now broadly adopted in the simulation of zinc proteins [19, 53, 54]. Due to the limited amount of cadmium‐bearing proteins in the PDB, the fitting of the new set of polarized (RESP) charges on the CYS and HIS coordinating residues, as well as the new σ and ϵ parameters for the Cd‐X interactions were integrated using QM calculations and QM/MM simulations as a reference. The fitting process led to the definition of three new types of cadmium‐coordinating residues, namely CYC (cysteine), and HDC (histidine ϵ), and HEC (histidine δ).

We tested our new FF on 10 cadmium‐bearing proteins taken from the PDB database by performing extensive molecular dynamics simulations. In all cases, the proposed FF was able to maintain during the MD simulation a stable tetrahedral coordination pattern and to accurately reproduce the experimental and QM/MM coordination distances. We also proved that our new FF for cadmium outperformed the AMBER standard force field both in reproducing the metal‐ligand distances and in stabilizing the coordination pattern.

Our new FF can be implemented at the input level on the most popular molecular dynamics packages with no code modification whatsoever and is compatible with the more recent AMBER FF14SB [24] and FF19SB [55].

This advancement can hopefully enable carrying out extensive and reliable classical simulations aimed at identifying structural rearrangements or aberrations that might shed light on the mechanisms of cadmium‐induced toxicity, in combination with experimental evidence.

Indeed, our new FF may find suitable employment in the refinement of NMR or X‐ray structures of cadmium‐binding proteins, whose active sites include one of the reparameterized amino acids.

## Supporting information


**Data S1**: RESP charges, atomic types, and Lennard‐Jones parameters for the CYC, HDC and HEC cadmium‐binding residues. RMSD fluctuations and interatomic Cd‐S and Cd‐N distance distributions obtained using the new FF and TIP3P water model. RMSD obtained from classical simulations (50 ns) with the new FF and with the AMBER standard one on seven cadmium‐bearing proteins (identified by the PBD codes 1DCD, 1FE0, 4C3D, 1VQ8 (chain 1), 1VQ8 (chain 3), 2JHF, 2PZI, and the 3 additional replicas of 2L1O).

Data S2.

## Data Availability

All data for reproducing the results presented in this study, starting structures, FF parameters, template input files, and ancillary software on HPC platforms for MD and QM/MM simulations using GROMACS and GROMACS/CP2K are available at the public repository Zenodo https://doi.org/10.5281/zenodo.14276548.
