# Supplementary material for: Development of a New AMBER Force Field for Cysteine and Histidine Cadmium‐Binding Proteins and Its Validation Through QM/MM MD Simulations
Source: J Comput Chem. 2025 Jun 13;46(16):e70154. doi: 10.1002/jcc.70154 (PMC12163698; doi:10.1002/jcc.70154)
Supplement: Supplementary file 1 — Data S1: RESP charges, atomic types, and Lennard‐Jones parameters for the CYC, HDC and HEC cadmium‐binding residues. RMSD fluctuations and interatomic Cd‐S and Cd‐N distance distributions obtained using the new FF and TIP3P water model. RMSD obtained from classical simulations (50 ns) with the new FF and with the AMBER standard one on seven cadmium‐bearing proteins (identified by the PBD codes 1DCD, 1FE0, 4C3D, 1VQ8 (chain 1), 1VQ8 (chain 3), 2JHF, 2PZI, and the 3 additional replicas of 2L1O). [file JCC-46-0-s002.pdf]

Supporting Information

Development of a New AMBER Force Field for Cysteine and Histidine Cadmium Binding Proteins and its Validation through QM/MM MD Simulations

Matteo Orlandi,[†,‡] Marina Macchiagodena,[†] Piero Procacci ,[*,†] Fabrizio Carta,[‡] Claudiu T. Supuran,[‡] and Marco Pagliai[*,†]

†*Dipartimento di Chimica "Ugo Schiff", Università degli Studi di Firenze, Via della Lastruccia 3, 50019 Sesto Fiorentino, Italy*

‡*Dipartimento di Neuroscienze, Psicologia, Area del Farmaco e Salute del Bambino, Università degli Studi di Firenze, Via Ugo Schiff 6, 50019 Sesto Fiorentino, Italy*

E-mail: piero.procacci@unifi.it; marco.pagliai@unifi.it

Table S1: New CYC residue upgraded atomic charges and comparison with both CYZ Macchiagodena *et al.*[1] FF for zinc metalloproteins and CYM AMBER standard FF for cysteinate residues.

Atom label	Atom type	New atom type	Atomic charges (e)		
			CYC	CYZ	CYM
N	N	N	-0.41570	-0.41570	-0.41570
H	H	H	0.27190	0.27190	0.27190
CA	CT	CT	-0.12880	-0.15350	-0.03510
HA	H1	H1	0.11700	0.07310	0.05080
CB	CT	CT	0.02030	0.09460	-0.24130
HB1	H1	H1	0.06970	0.05140	0.11220
HB2	H1	H1	0.06970	0.05140	0.11220
SG	SH	SC	-1.03350	-1.00260	-0.88440
C	C	C	0.59730	0.59730	0.59730
O	O	O	-0.56790	-0.56790	-0.56790

Table S2: New HEC residue upgraded atomic charges and comparison with both HEZ Macchiagodena *et al.*[1] FF for zinc metalloproteins and HIE AMBER standard FF histidine residues (Nδ is the coordinating atom).

Atom label	Atom type	New atom type	Atomic charges (e)		
			HEC	HEZ	HIE
N	N	N	-0.41570	-0.41570	-0.41570
H	H	H	0.27190	0.27190	0.27190
CA	CT	CT	0.60550	0.36920	-0.05810
HA	H1	H1	-0.08550	0.00010	0.13600
CB	CT	CT	-0.28930	-0.14090	-0.00740
HB1	HC	HC	0.09060	0.04750	0.03670
HB2	HC	HC	0.09060	0.04750	0.03670
CG	CC	CC	0.32490	0.52290	0.18680
ND1	NB	NC4	-1.30940	-1.20080	-0.54320
CE1	CR	CR	0.49010	0.28080	0.16350
HE1	H5	H5	0.07620	0.12280	0.14350
NE2	NA	NA	-0.12750	-0.05660	-0.27950
HE2	H	H	0.21890	0.23360	0.33390
CD2	CW	CW	-0.05140	-0.21730	-0.22070
HD2	H4	H4	0.08070	0.10560	0.18620
C	C	C	0.59730	0.59730	0.59730
O	O	O	-0.56790	-0.56790	-0.56790

Table S3: New HDC residue upgraded atomic charges and comparison with both HDZ Macchiagodena *et al.*[1] FF for zinc metalloproteins and HID AMBER standard FF histidine residues (Nε is the coordinating atom).

Atom label	Atom type	New atom type	Atomic charges (e)		
			**HDC**	**HDZ**	**HID**
N	N	N	-0.41570	-0.41570	-0.41570
H	H	H	0.27190	0.27190	0.27190
CA	CT	CT	0.36710	0.36670	0.01880
HA	H1	H1	0.01330	-0.04520	0.08810
CB	CT	CT	-0.26260	-0.26500	-0.04620
HB1	HC	HC	0.07580	0.09110	0.04020
HB2	HC	HC	0.07580	0.09110	0.04020
CG	CC	CC	-0.01940	0.08800	-0.02660
ND1	NA	NA	0.08510	-0.08020	-0.38110
HD1	H	H	0.12490	0.22530	0.36490
CE1	CR	CR	0.22140	0.22300	0.20570
HE1	H5	H5	0.19410	0.14640	0.13920
NE2	NB	NC5	-1.29200	-1.01850	-0.57270
CD2	CV	CV	0.55660	0.18380	0.12920
HD2	H4	H4	-0.02570	0.10790	0.11470
C	C	C	0.59730	0.59730	0.59730
O	O	O	-0.56790	-0.56790	-0.56790

Table S4: New σ and the standard ε parameters designed for the Cd coordinating atoms: S, renamed SC and N, renamed NC4(Nδ) or NC5(Nε). The comparisons with the previously developed new atom types (SZ and NZ) for the Zn(II) FF[1] and also with those belonging to the AMBER standard FF[2] are reported.

Atom type	σ new FF(Å)	σ Zn(Å)	σ AMBER(Å)	ε(kJ/mol)
Cd	2.51590	/	2.51590	$7.41997 \cdot 10^{-2}$
SC	4.40000	3.66359	3.56359	1.04600
NC4	4.20000	3.35000	3.25000	$7.11280 \cdot 10^{-1}$
NC5	3.95000	3.35000	3.25000	$7.11280 \cdot 10^{-1}$

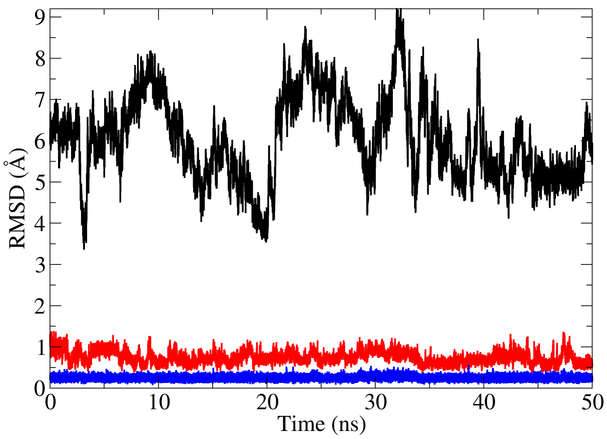

(a) 4C3D (3C1H)-tip3p

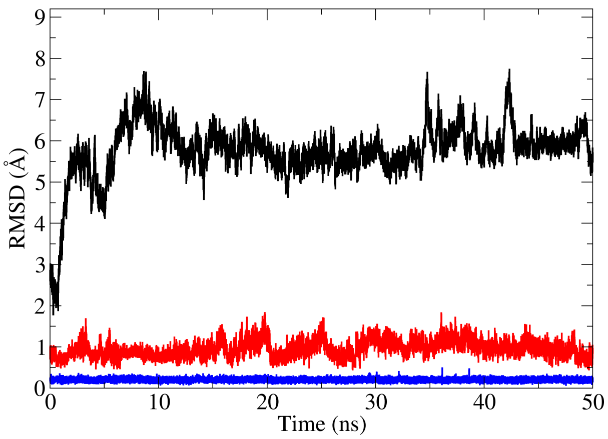

(b) 4C3D (3C1H)-Spc/e

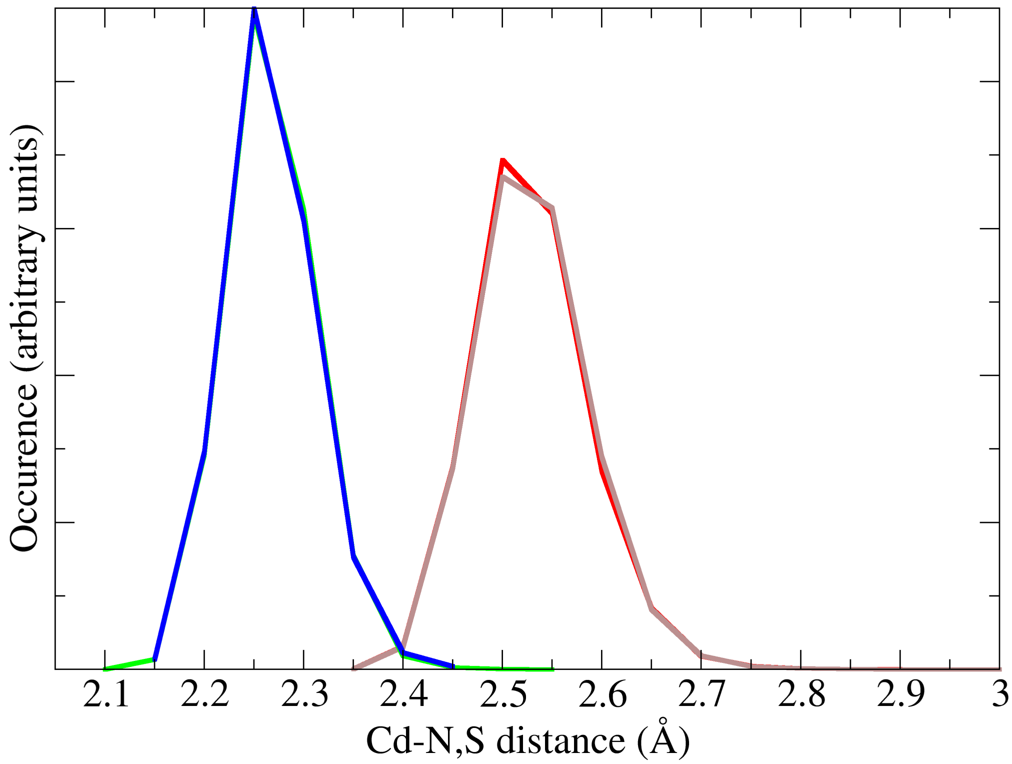

(c) 4C3D (3C1H)

Figure S1: RMSD fluctuations and interatomic Cd-S and Cd-N distance distributions obtained through MDs of the protein **4C3D** (new FF). In the top panel, we show the comparison between the RMSD fluctuations obtained by employing the tip3p water model (a) and the spc/e one (b). In the bottom panel (c), we report the comparison between the interatomic distance distributions obtained through the tip3p water model Cd-S (maroon) and Cd-N (green), with those calculated with the spc/e one: Cd-S in red and Cd-N in blue.

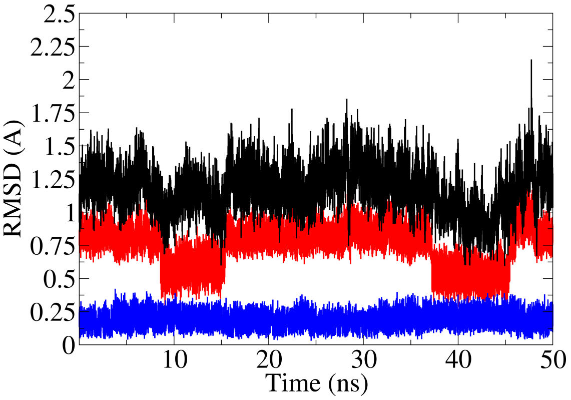

(a) 1R0I (4C)-tip3p

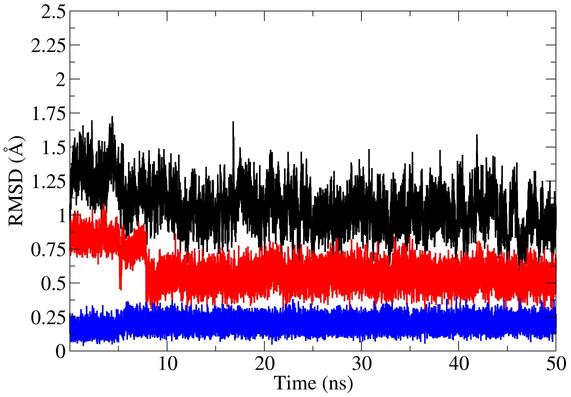

(b) 1R0I (4C)-Spc/e

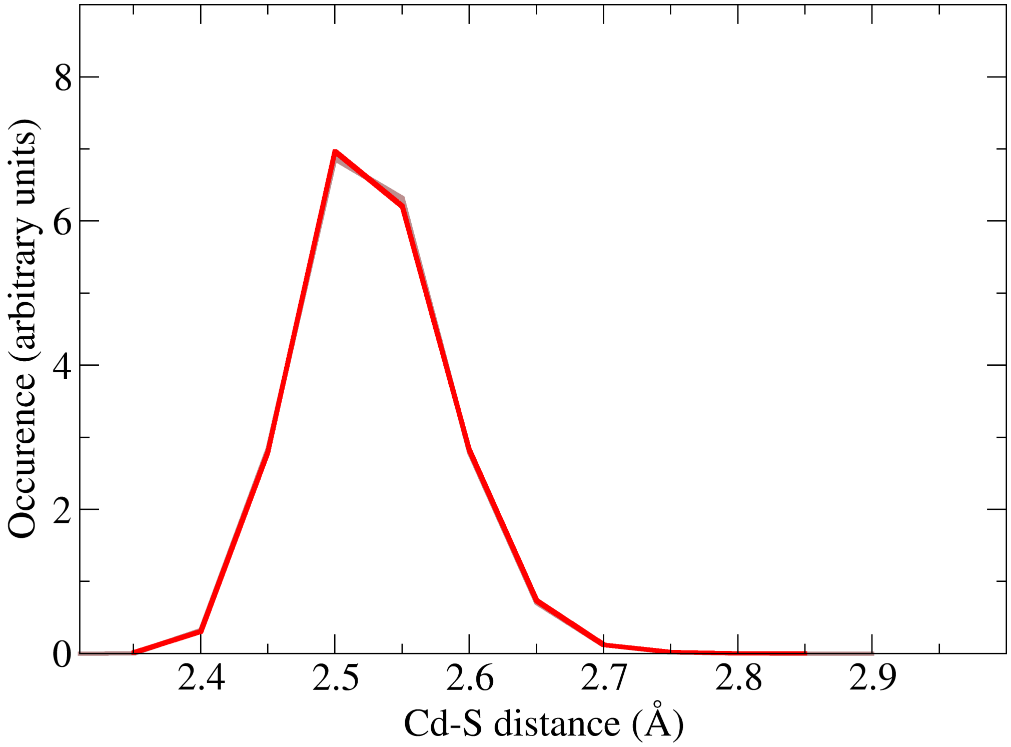

(c) 1R0I (4C)

Figure S2: RMSD fluctuations and interatomic Cd-S and Cd-N distance distributions obtained through MDs of the protein **1R0I** (new FF). In the top panel, we show the comparison between the RMSD fluctuations obtained by employing the tip3p water model (a) and the spc/e one (b). In the bottom panel (c), we report the comparison between the interatomic distance distributions obtained through the tip3p water model Cd-S (maroon) and Cd-N (green), with those calculated with the spc/e one: Cd-S in red and Cd-N in blue.

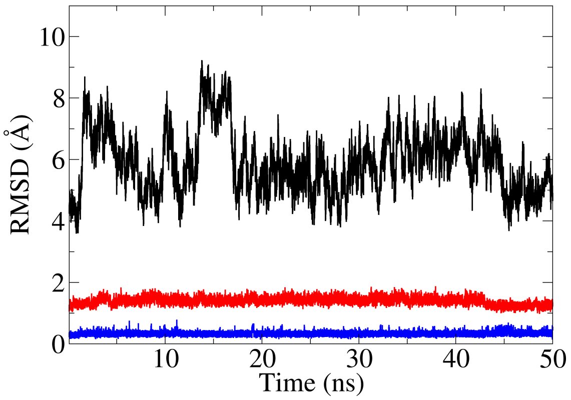

(a) 2L1O (2C2H)-tip3p

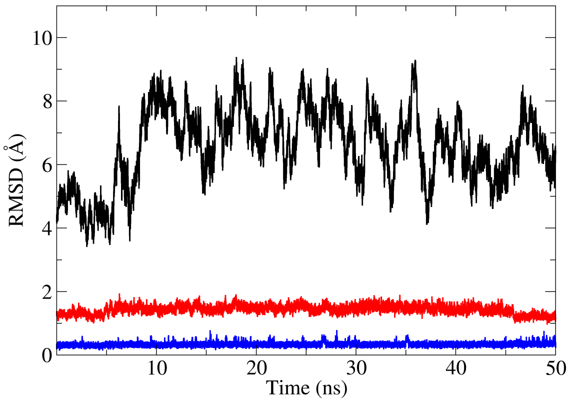

(b) 2L1O (2C2H)-Spc/e

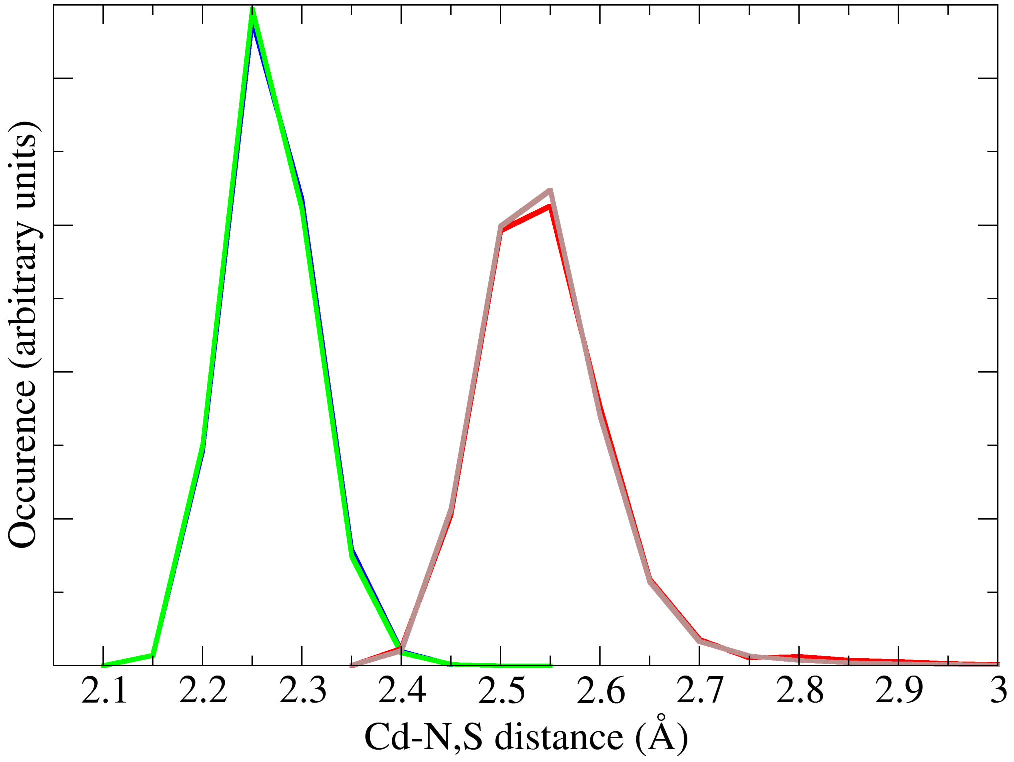

(c) 2L1O (2C2H)

Figure S3: RMSD fluctuations and interatomic Cd-S and Cd-N distance distributions obtained through MDs of the protein **2L1O** (new FF). In the top panel, we show the comparison between the RMSD fluctuations obtained by employing the tip3p water model (a) and the spc/e one (b). In the bottom panel (c), we report the comparison between the interatomic distance distributions obtained through the tip3p water model Cd-S (maroon) and Cd-N (green), with those calculated with the spc/e one: Cd-S in red and Cd-N in blue.

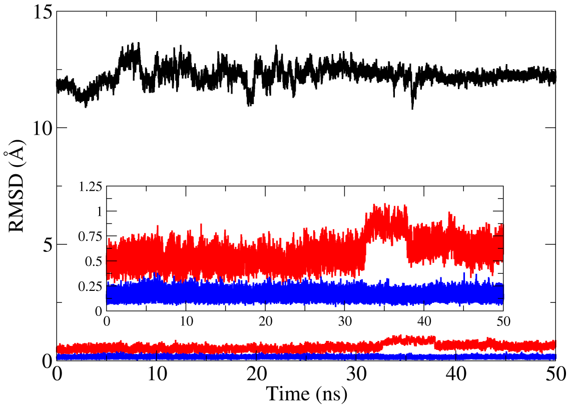

(a) $1VQ8_1$ (4C)-tip3p

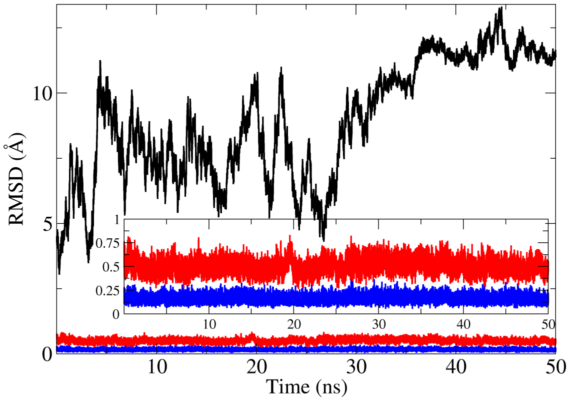

(b) $1VQ8_1$ (4C)-Spc/e

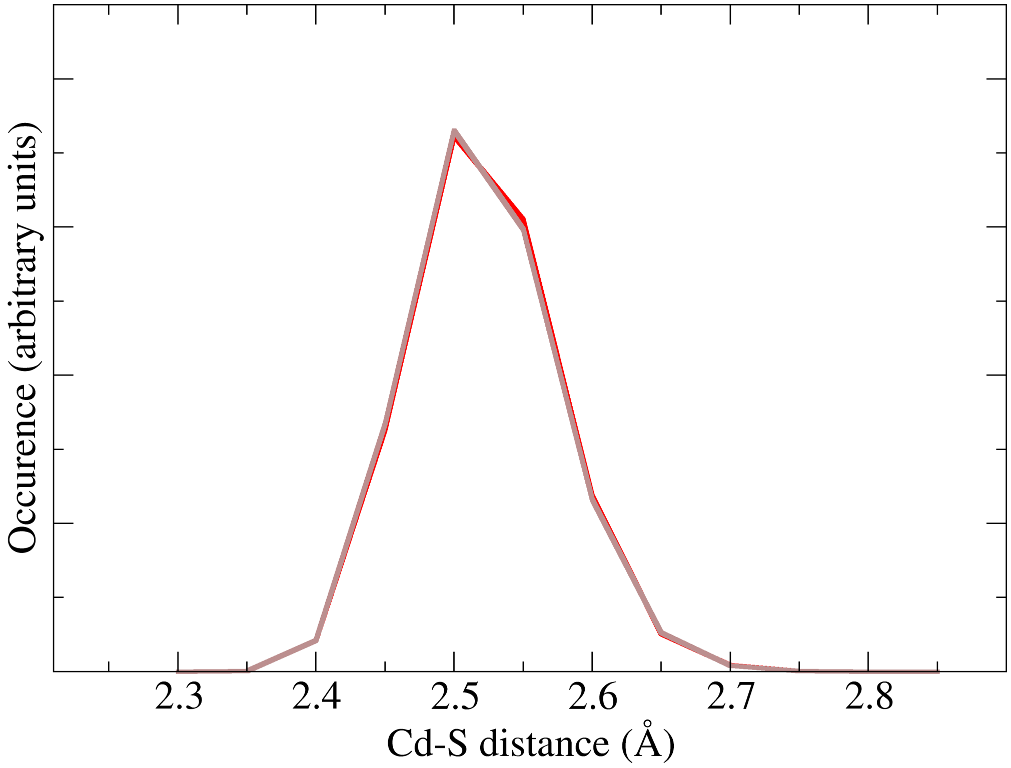

(c) $1VQ8_1$ (4C)

Figure S4: RMSD fluctuations and interatomic Cd-S and Cd-N distance distributions obtained through MDs of the protein **$1VQ8_1$**(new FF). In the top panel, we show the comparison between the RMSD fluctuations obtained by employing the tip3p water model (a) and the spc/e one (b). In the bottom panel (c), we report the comparison between the interatomic distance distributions obtained through the tip3p water model Cd-S (maroon) and Cd-N (green), with those calculated with the spc/e one: Cd-S in red and Cd-N in blue.

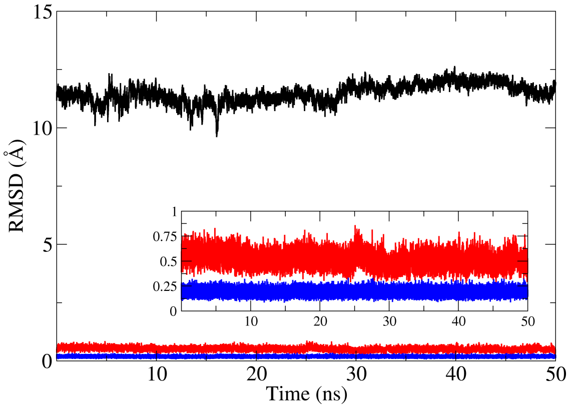

(a) 1VQ8$_3$ (4C)-tip3p

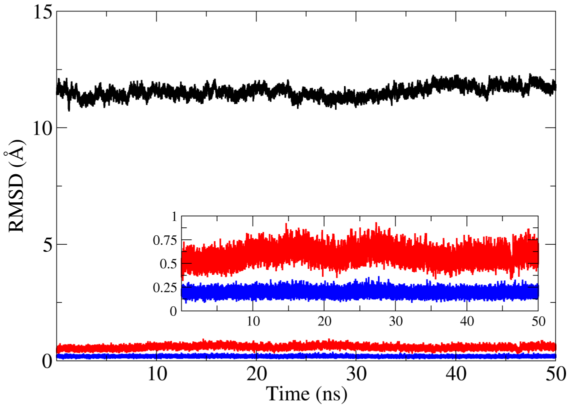

(b) 1VQ8$_3$ (4C)-Spc/e

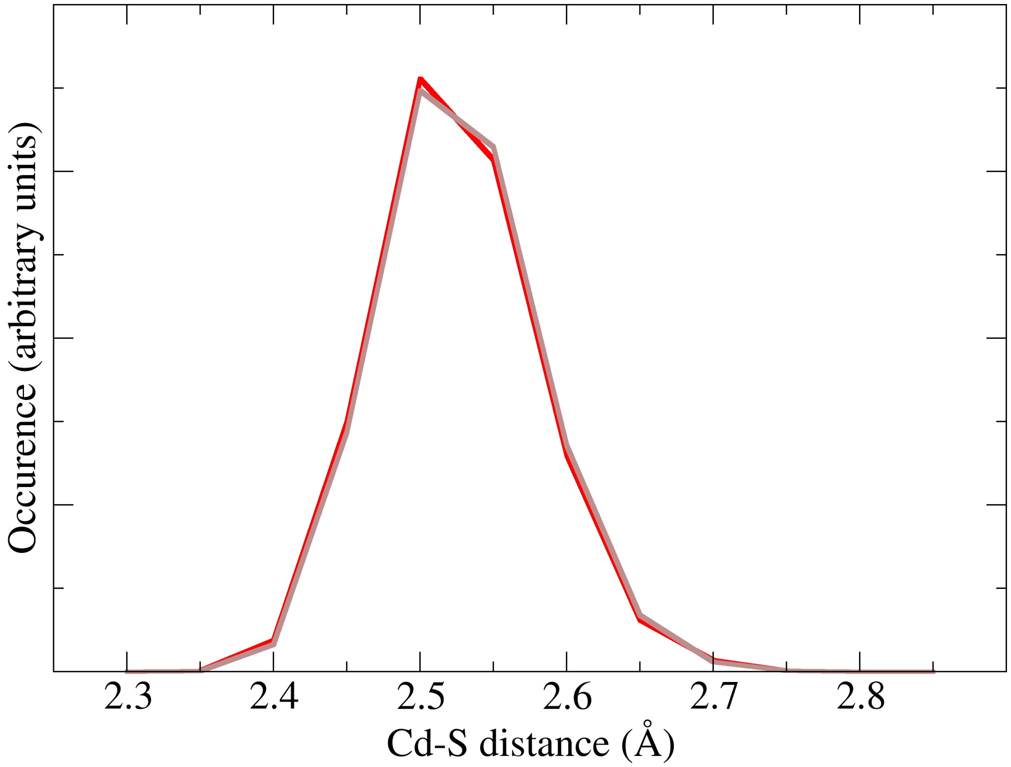

(c) 1VQ8$_3$ (4C)

Figure S5: RMSD fluctuations and interatomic Cd-S and Cd-N distance distributions obtained through MDs of the protein **1VQ8**$_3$(new FF). In the top panel, we show the comparison between the RMSD fluctuations obtained by employing the tip3p water model (a) and the spc/e one (b). In the bottom panel (c), we report the comparison between the interatomic distance distributions obtained through the tip3p water model Cd-S (maroon) and Cd-N (green), with those calculated with the spc/e one: Cd-S in red and Cd-N in blue.

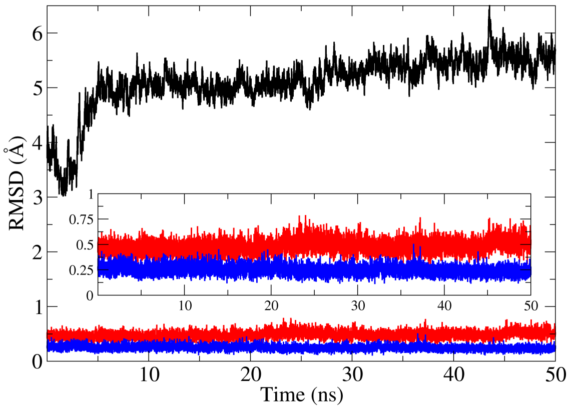

(a) 2PZI (4C)-tip3p

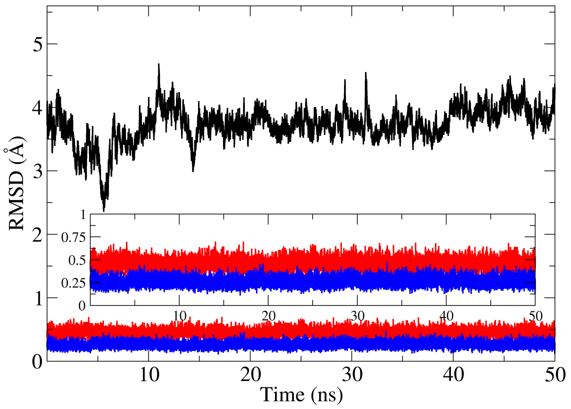

(b) 2PZI (4C)-Spc/e

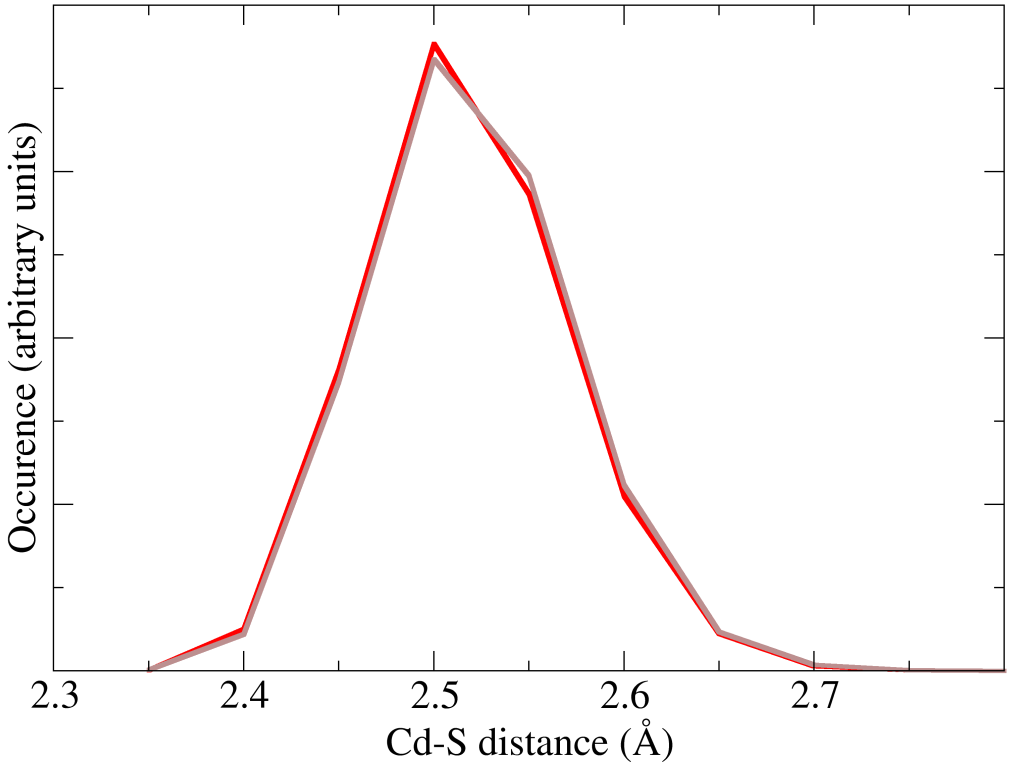

(c) 2PZI (4C)

Figure S6: RMSD fluctuations and interatomic Cd-S and Cd-N distance distributions obtained through MDs of the protein **2PZI**(new FF). In the top panel, we show the comparison between the RMSD fluctuations obtained by employing the tip3p water model (a) and the spc/e one (b). In the bottom panel (c), we report the comparison between the interatomic distance distributions obtained through the tip3p water model Cd-S (maroon) and Cd-N (green), with those calculated with the spc/e one: Cd-S in red and Cd-N in blue.

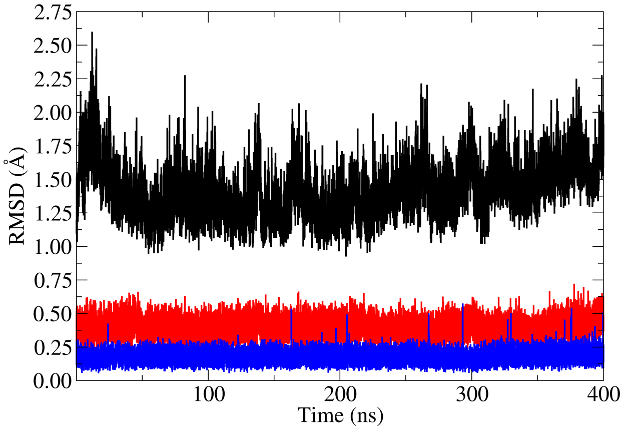

(a) 1IPP (3C1H) chain A

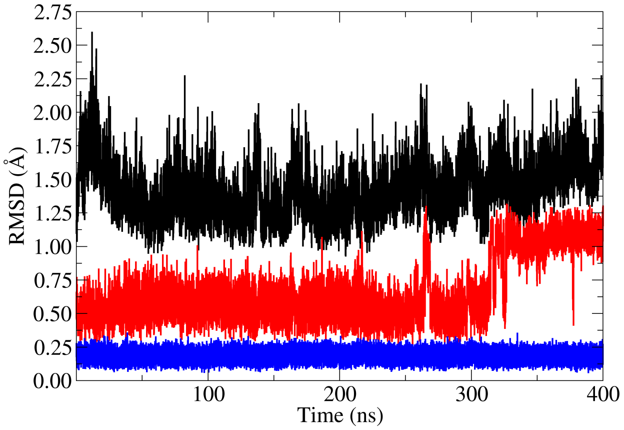

(b) 1IPP (3C1H) chain B

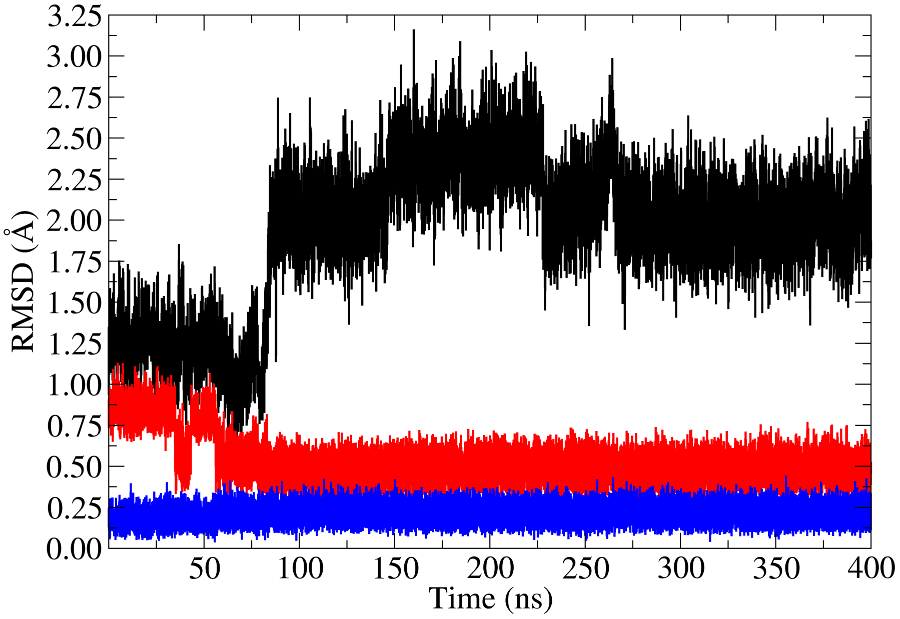

(c) 1R0I (4C)

Figure S7: RMSD fluctuations over **1IPP** (chain A panel a and chain B panel b) and **1R0I** (panel c) 400 ns MD simulations (new FF and spc/e water). In blue: Cd-coordinating atoms, in red Cd-coordinating residues and in black backbone

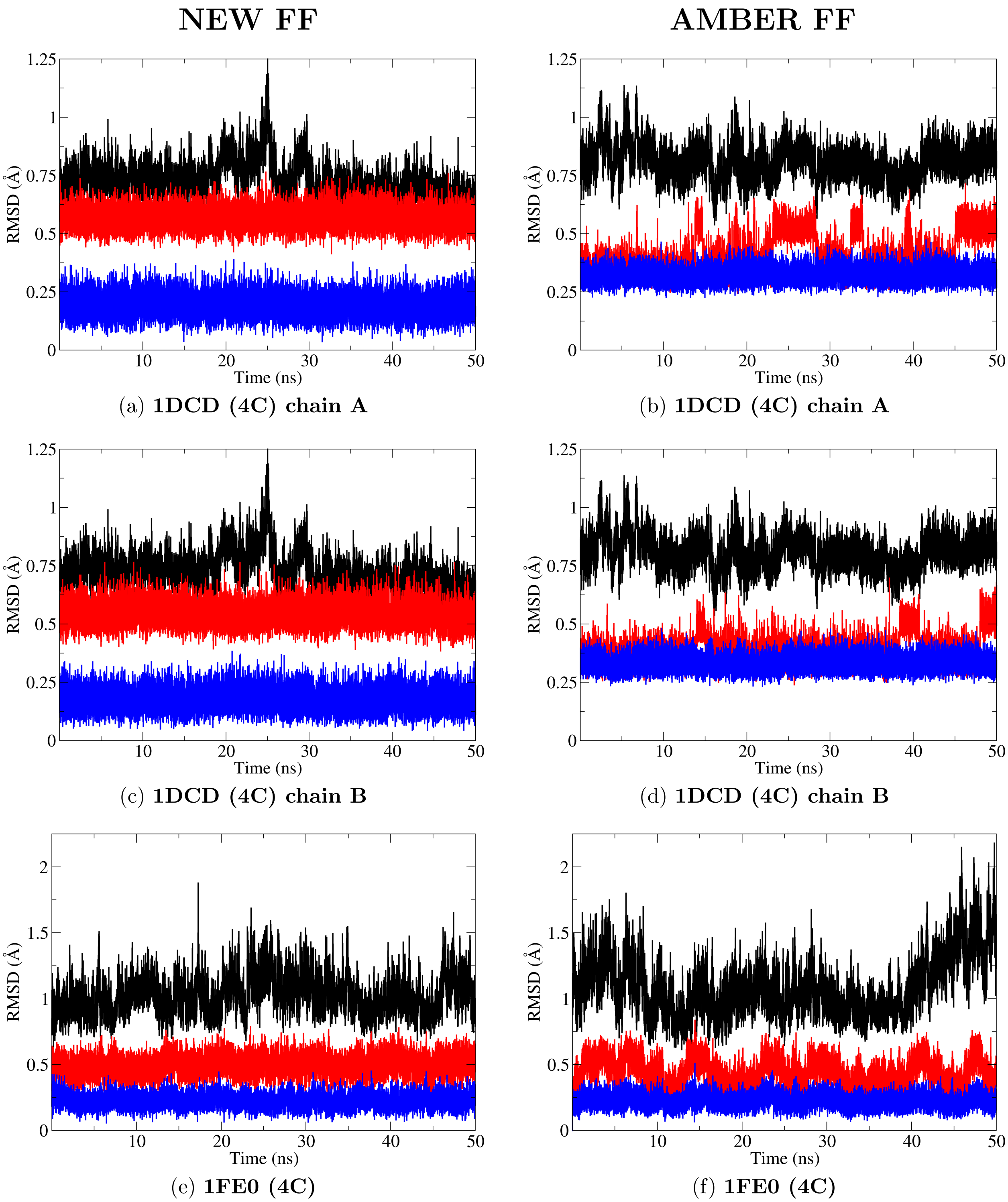

Figure S8: From top to bottom:**1DCD** (2 site 4C, one site in chain A, panels a-b and the other one in chain B, panels c-d) and **1FE0** (4C) panels e-f. RMSD fluctuations: in blue: Cd-coordinating atoms, in red Cd-coordinating residues and in black backbone. On the left column are shown the results obtained through our new FF whereas on the right one those of the AMBER FF. For 1DCD chains the black line is referred to the whole protein backbone.

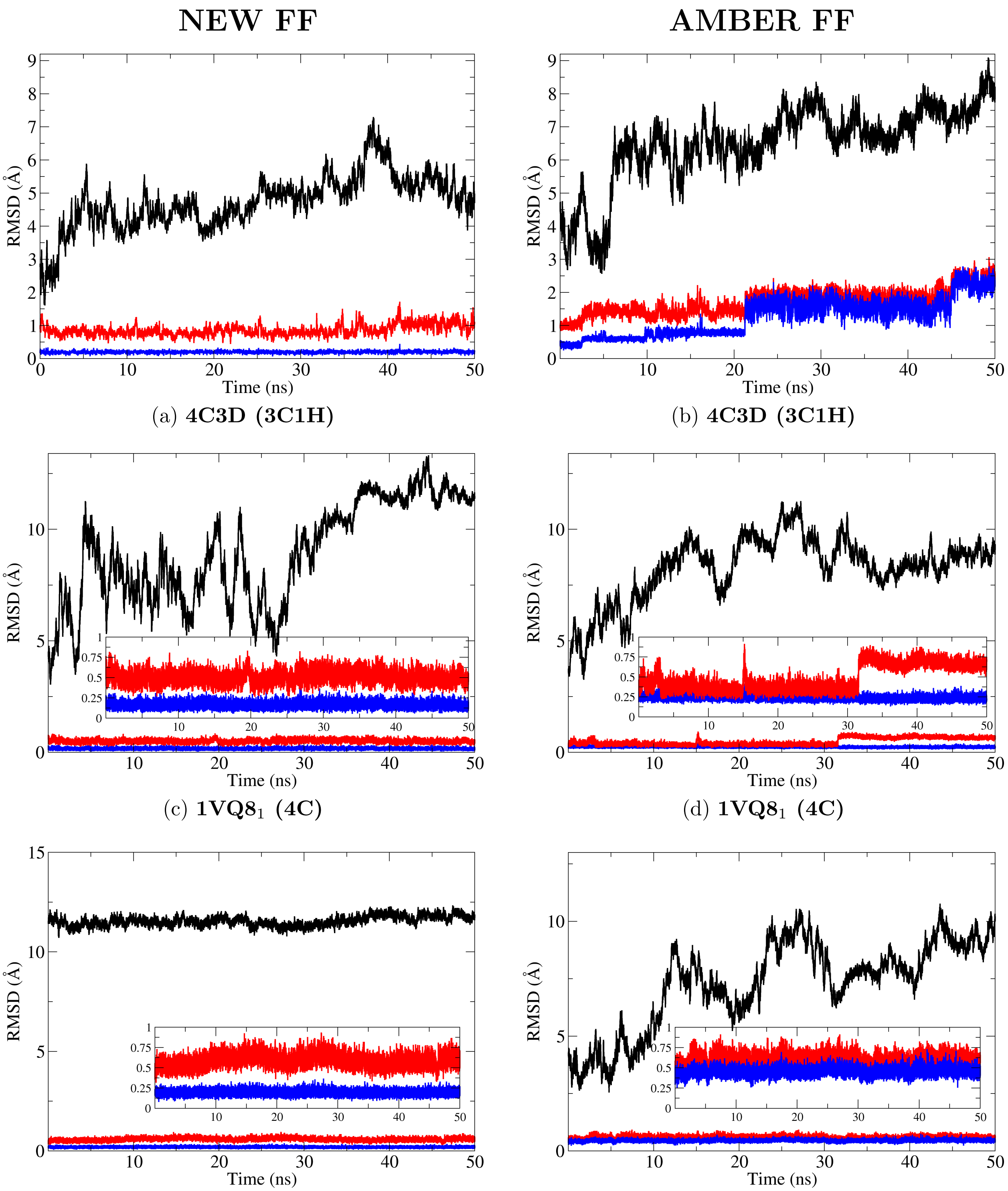

(a) **4C3D (3C1H)** (b) **4C3D (3C1H)**

(c) **1VQ8**$_1$ **(4C)** (d) **1VQ8**$_1$ **(4C)**

(e) **1VQ8**$_3$ **(4C)** (f) **1VQ8**$_3$ **(4C)**

Figure S9: From top to bottom: **4C3D** (3C1H, Nε) panels a-b, **1VQ8**$_1$ (4C) panels c-d and **1VQ8**$_3$ (4C) panels e-f. RMSD fluctuations: in blue: Cd-coordinating atoms, in red Cd-coordinating residues and in black backbone. On the left column are shown the results obtained through our new FF whereas on the right one those of the AMBER FF.

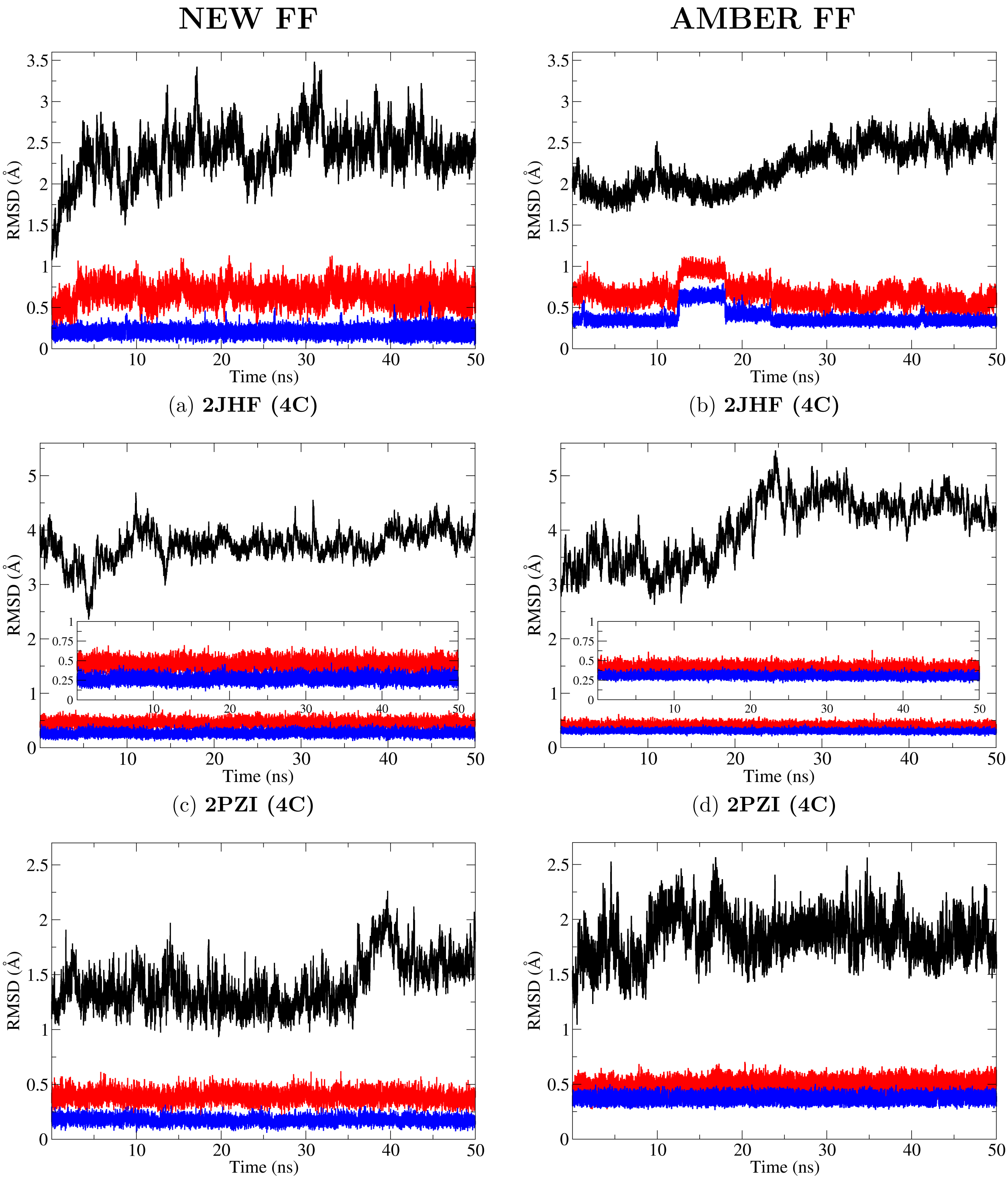

(a) **2JHF (4C)** (b) **2JHF (4C)**
(c) **2PZI (4C)** (d) **2PZI (4C)**
(e) **1IPP (3C1H)** (f) **1IPP (3C1H)**

Figure S10: From top to bottom: **2JHF** (4C) panels a-b, **2PZI** (4C) panels c-d and **1IPP** (3C1H, chain A) panels e-f. RMSD fluctuations: in blue: Cd-coordinating atoms, in red Cd-coordinating residues and in black backbone. On the left column are shown the results obtained through our new FF whereas on the right one those of the AMBER FF.

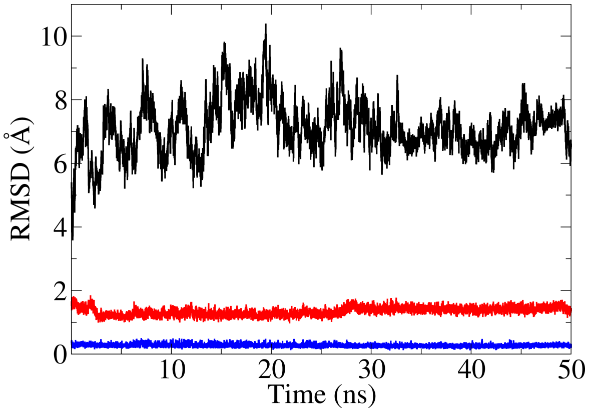

(a)

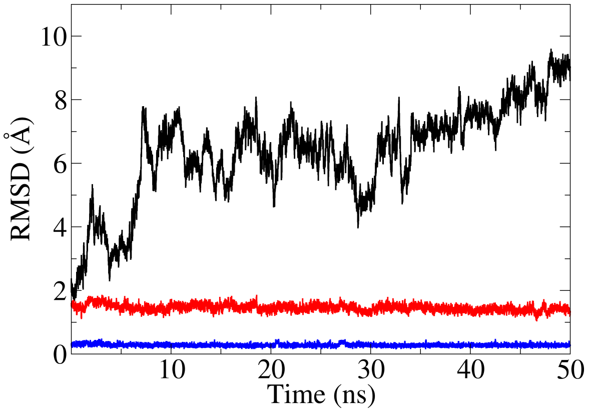

(b)

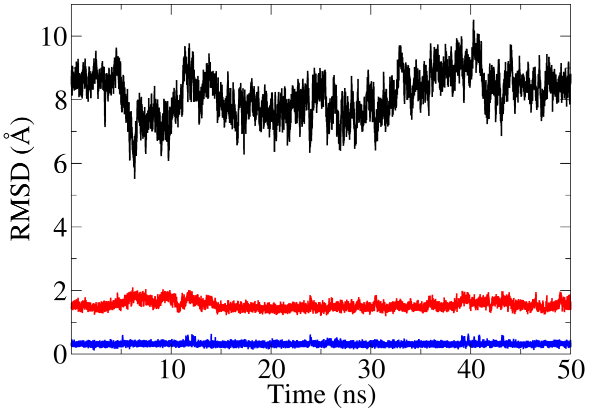

(c)

Figure S11: panels a-c: RMSD fluctuations of three 50 ns MD simulations replica (new FF) of **2L1O** protein. In blue: Cd-coordinating atoms, in red Cd-coordinating residues and in black backbone.

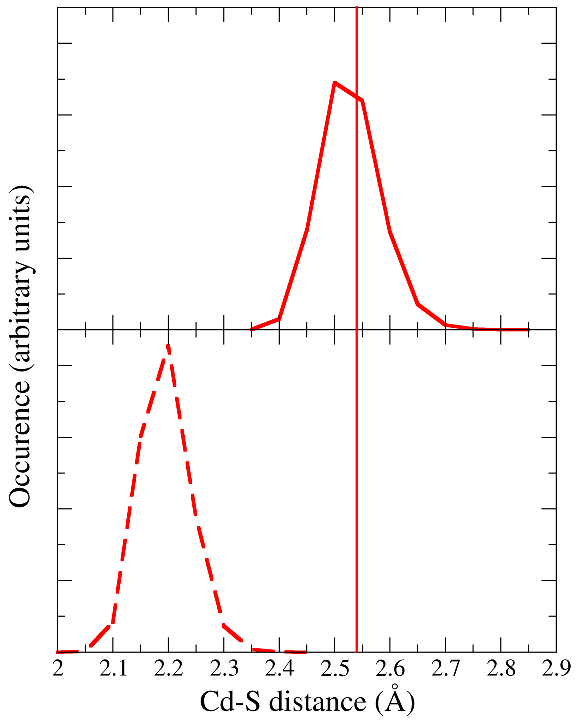

(a) 1DCD (4C)

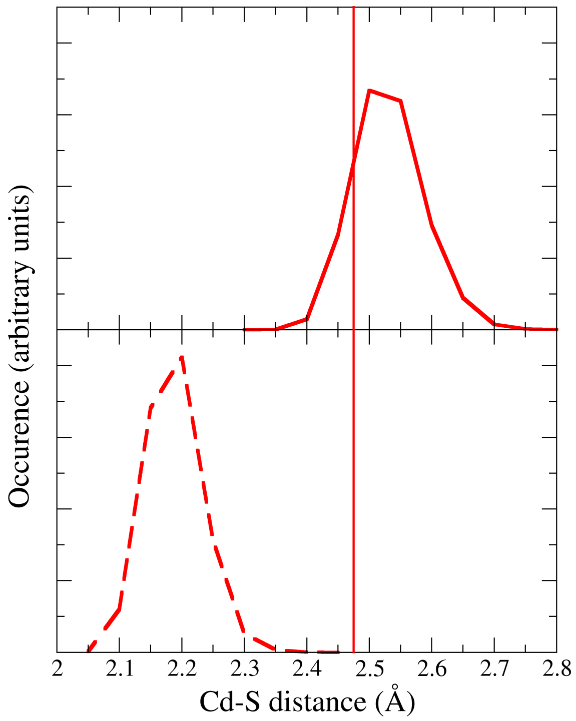

(b) 1FE0 (4C)

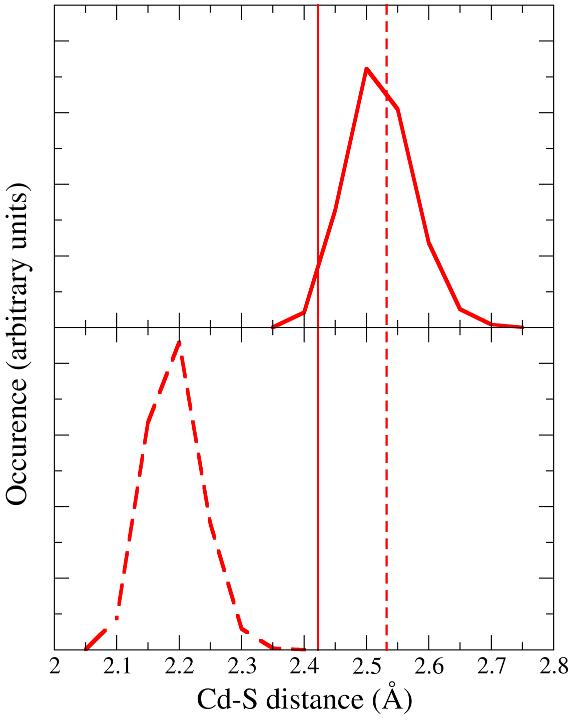

(c) $1VQ8_1$ (4C)

Figure S12: Interatomic Cd-S distance distributions obtained through MDs of the proteins 1DCD (a), 1FE0 (b) and $1VQ8_1$ (c). In top and bottoms panels we report the results obtained with the new (solid line) and AMBER standard FFs (dashed line), respectively. The solid vertical lines are the X-ray experimental distances while the dashed ones indicate the PDB-redo analogous.

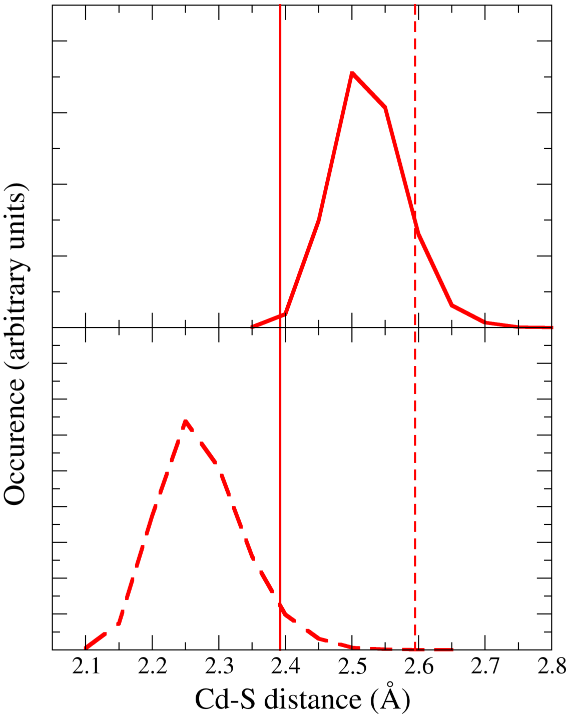

(a) $1VQ8_3$ (4C)

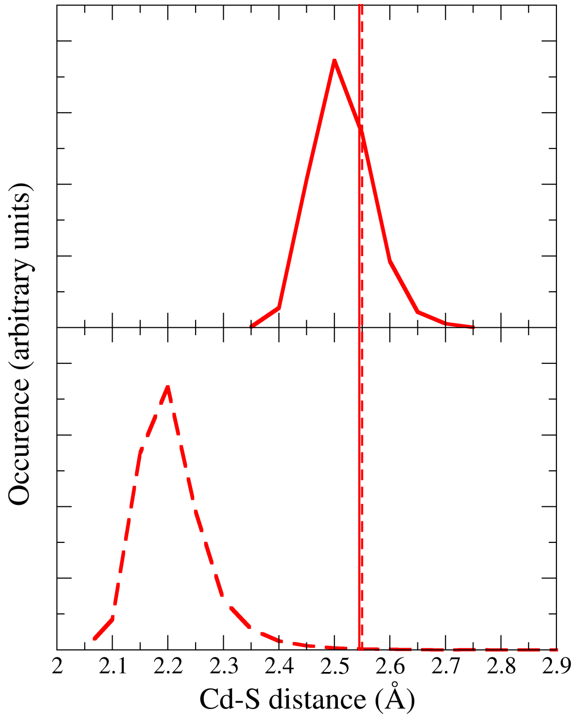

(b) 2JHF (4C)

Figure S13: Interatomic Cd-S distance distributions obtained through MDs of the proteins $1VQ8_3$ (a) and 2JHF (b). In top and bottoms panels we report the results obtained with the new (solid line) and AMBER standard FFs (dashed line), respectively. The solid vertical lines are the X-ray experimental distances while the dashed ones indicate the PDB-redo analogous.

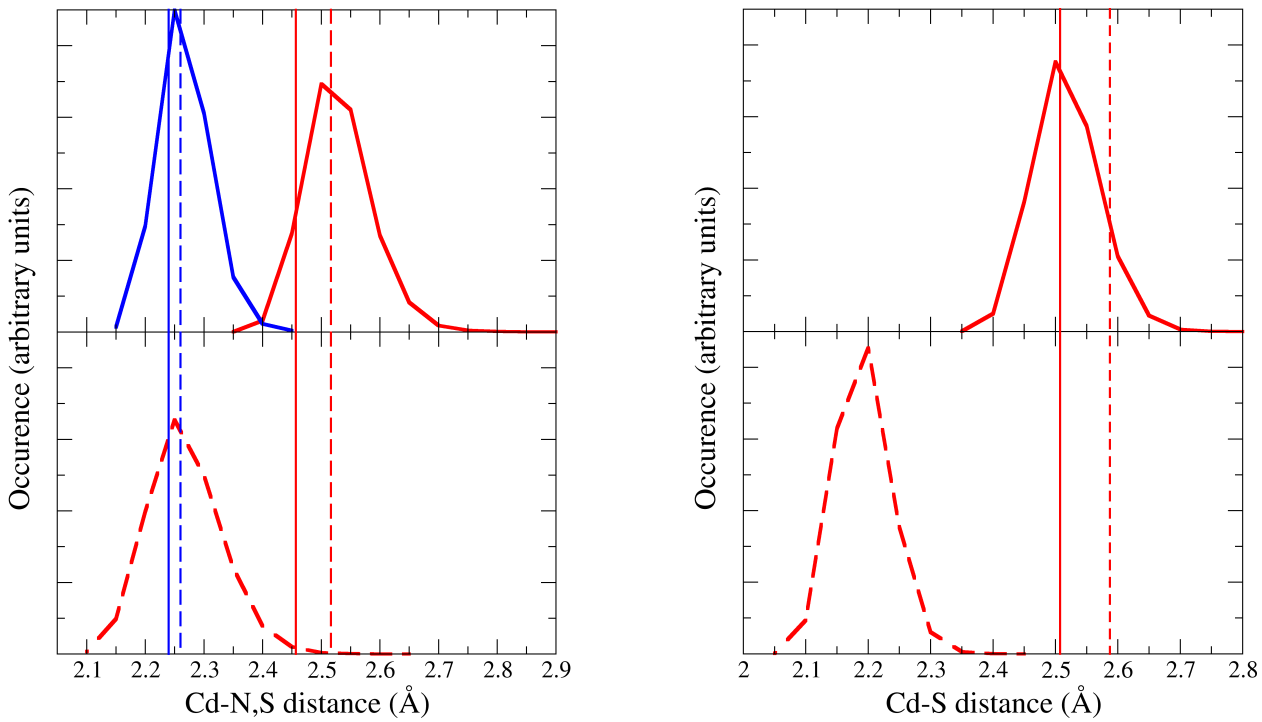

(a) 4C3D (3C1H)

(b) 2PZI (4C)

Figure S14: Interatomic distance distributions obtained through MDs of the proteins 4C3D (a) and 2PZI (b). In top and bottoms panels we report the results obtained with the new (solid line) and AMBER standard FFs (dashed line), respectively. The red and blue colors refer to the Cd-S and Cd-N distance distribution, respectively. The solid vertical lines are the X-ray experimental distances while the dashed ones indicate the PDB-redo analogous. For 4C3D protein, the interatomic Cd-N distance distribution of the detached histidine residue is not reported.

References

(1) Macchiagodena, M.; Pagliai, M.; Andreini, C.; Rosato, A.; Procacci, P. Upgrading and Validation of the AMBER Force Field for Histidine and Cysteine Zinc(II)-Binding Residues in Sites with Four Protein Ligands. *J. Chem. Inf. Model.* **2019**, *59*, 3803–3816.

(2) Li, P.; Merz, K. M. J. Taking into Account the Ion-Induced Dipole Interaction in the Nonbonded Model of Ions. *J. Chem. Theory Comput.* **2014**, *10*, 289–297.
